# Removing the no-analogue bias in modern accelerated tree growth leads to stronger medieval drought

**DOI:** 10.1038/s41598-019-39040-5

**Published:** 2019-02-21

**Authors:** Tobias Scharnweber, Karl-Uwe Heußner, Marko Smiljanic, Ingo Heinrich, Marieke van der Maaten-Theunissen, Ernst van der Maaten, Thomas Struwe, Allan Buras, Martin Wilmking

**Affiliations:** 1 Institute of Botany and Landcape Ecology, University of Greifswald, 17487 Greifswald, Germany; 2 German Archaeological Institute DAI, 14195 Berlin, Germany; 3 German Research Centre for Geosciences GFZ, 14473 Potsdam, Germany; 4 Present Address: Geography Department, Humboldt-University Berlin, 12489 Berlin, Germany; 5 Institute of Forest Growth and Forest Computer Sciences, Technical University Dresden, 01737 Tharandt, Germany; 6 Forest Ecology and Forest Management, Wageningen University and Research, 6708 PB Wageningen, The Netherlands; 7 Present Address: Land-Surface-Atmosphere-Interactions, Technische Universität München, 85354 Freising, Germany

## Abstract

In many parts of the world, especially in the temperate regions of Europe and North-America, accelerated tree growth rates have been observed over the last decades. This widespread phenomenon is presumably caused by a combination of factors like atmospheric fertilization or changes in forest structure and/or management. If not properly acknowledged in the calibration of tree-ring based climate reconstructions, considerable bias concerning amplitudes and trends of reconstructed climatic parameters might emerge or low frequency information is lost. Here we present a simple but effective, data-driven approach to remove the recent non-climatic growth increase in tree-ring data. Accounting for the no-analogue calibration problem, a new hydroclimatic reconstruction for northern-central Europe revealed considerably drier conditions during the medieval climate anomaly (MCA) compared with standard reconstruction methods and other existing reconstructions. This demonstrates the necessity to account for fertilization effects in modern tree-ring data from affected regions before calibrating reconstruction models, to avoid biased results.

## Introduction

Proxies are a scientific bridge to infer climate variability beyond the instrumental period. Tree-rings with their widespread distribution, annual resolution in long continuous records, straightforward translation into climate variables and absolute dating to the calendar year, constitute one of the most important and influential climate proxy sources over the late Holocene^1,2^. Nevertheless, rather than being precise gauges of temperature or rainfall, measures of tree-ring parameters, as with all biogenic proxies used for climate reconstructions, are multichannel recorders that integrate information from a multitude of processes in one value. In dendroclimatology we then try to extract the climatic component involved in the chronological variance of parameters like annual tree-ring width (TRW), maximum latewood density (MXD), stable isotope composition or wood anatomical features. When calibrating tree-ring derived proxy data against single climate parameters, explained variances rarely exceed 50% (especially in temperate environments); leaving more than half of the variance as unexplained noise^3^. This noise component needs to be properly addressed in order to avoid misinterpretation of temporal trends. Some general problems arise here: First, the contemporary growth environment with for example peak CO_2_ concentrations and novel atmospheric deposition rates of pollutants like Sulphur compounds or fertilizers like wet and dry Nitrogen depositions, is unmatched by past conditions. As a consequence of this, tree growth rates have accelerated at some places^4–8^ or have been (temporarily) depressed at other sites with heavy atmospheric pollution loads due to soil acidification or direct detrimental influence of the pollutants^9,10^. This creates a so called no-analogue problem^11^, which, according to the uniformitarian principle, needs to be considered, if we want to continue using the “present as a key to the past”^12^. Second, biological organisms like trees and shrubs are living entities and as such undergo a constant process of adaptation that may result in a change in reaction to the same climatic conditions over time (see for example the discussion on increasing water use efficiency which is also connected to CO_2_ rise and N-deposition)^13,14^. In our study we specifically address these problems and show that 1) a better separation of non-climatic noise from climatic signals in tree-ring data is possible, which 2) significantly changes reconstructed climatic amplitudes.

In dendroclimatology the problem of divergent average growth rates between historical and contemporary tree-ring material is a known phenomenon that hampers an uniform statistical treatment of the different data sources^15–18^. However, climate reconstructions attempting to preserve as much low frequency variability as possible usually use one common detrending curve; the so called regional curve (RC) that is applied to the raw TRW or wood density data to remove the inherent age trend^19–22^. The basic assumption behind a RC is that in a given climatically defined region, species specific general growth curves exist (mostly of a negative exponential form), which mirror the decline of TRW with age and size. Deviations from this curve are interpreted as resulting from environmental drivers such as changes in climate. The RC is thereby directly derived from the raw data and requires some homogeneity of the original dataset^19,20^. If the different data sources like subfossil stems, historic timber and living wood samples that finally are combined into one chronology differ too strongly in their mean and/or variance, either individual detrending curves are fitted to the raw data series or a split RC detrending is used as an alternative^15,19–21^. However, individual detrending limits the low frequency information to the maximum individual series length (or maximum tree-age), the so-called “segment-length curse”^23^. While separate regional curve standardization (RCS) of the subfossil or historic wood sources and of the living material can preserve more of the centennial trends, it will inevitably lead to the loss of absolute amplitudes and some lower frequency variation^21^. Applied to reconstructions, both statistical treatments therefore impede a direct comparison (in °C or mm rainfall) of the instrumental period with the preceding centuries to millennia.

In addition to unprecedented levels of greenhouse gases and atmospheric fertilization and pollution, different forest management practices, large scale disturbances and the degree of habitat opening are thought to bias longer term trends in modern tree-ring data^17,24^. Therefore, the use of one common RCS for historical/subfossil and modern tree-ring data is regarded as inappropriate in regions with a strong anthropogenic imprint^15,17^. The empirical identification and removal of these non-climatic trends is however possible. We demonstrate this by applying a trend detection and removal algorithm to a highly replicated composite TRW-dataset of historical and modern beech (*Fagus sylvatica* L.) wood and succeed to place 1000 years of hydroclimatic variability for a densely cultivated region of northern central-Europe in a modern context, putting a special focus on absolute differences between the MCA and modern times.

## Results and Discussion

### Accounting for the no-analogue situation

**Figure 1:**
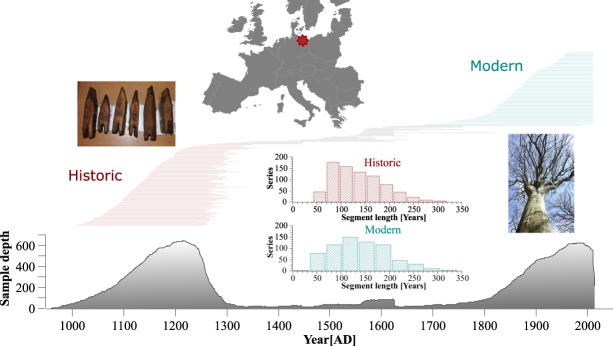
Location of the study sites in NE-Germany and segment plots of series distribution and sample depth together with histograms of the segment length (age) for the similarly highly replicated historic (980–1300 AD) and modern (1700–2014 AD) parts of the dataset. Compared with the modern trees the age structure of the historic part is slightly skewed towards younger trees.

The historical part of our dataset consists of 923 series from beech construction wood, mainly radially split planks, collected over the recent decades by the German Archaeological Institute (Fig. 1). Compared with oak, a classical construction wood and a chief species in European dendroclimatology^25^, beech wood offers two advantages when used for climate reconstructions. First, it was also locally available in medieval times and in contrast to oak not traded long distances. This assures a regionally well-defined climate signal in its TRW pattern. Second, over the period of instrumental data, RW of beech shows consistent and temporally stable correlations to summer moisture availability, irrespective of the micro-site conditions the trees grew in (wet/dry)^26,27^. The living part of the dataset consists of 973 series from the same region as the historical wood, covering a variety of site conditions and forest types including unmanaged nature reserves. Widespread cutting activities during and after World War II are exhibited in the TRW data of the living trees as clustered growth releases. Therefore a disturbance analysis was conducted and all series showing clear signs of this non-climatic growth increase (297 series) were removed from the original dataset. The final dataset contains 1599 series and is similarly highly replicated during the historic (980–1300 AD; 783 series) and modern (1700–2014 AD; 676 series) periods. Low construction activities, after ~1350 AD^28^ and/or abandonment of beech wood in constructions account for the low replication between 1300 and 1700 AD (Fig. 1). To account for a potential bias due to this low replication, we focus in our results on the comparison of the highly replicated modern warm period (MOWP) with the MCA.

**Figure 2:**
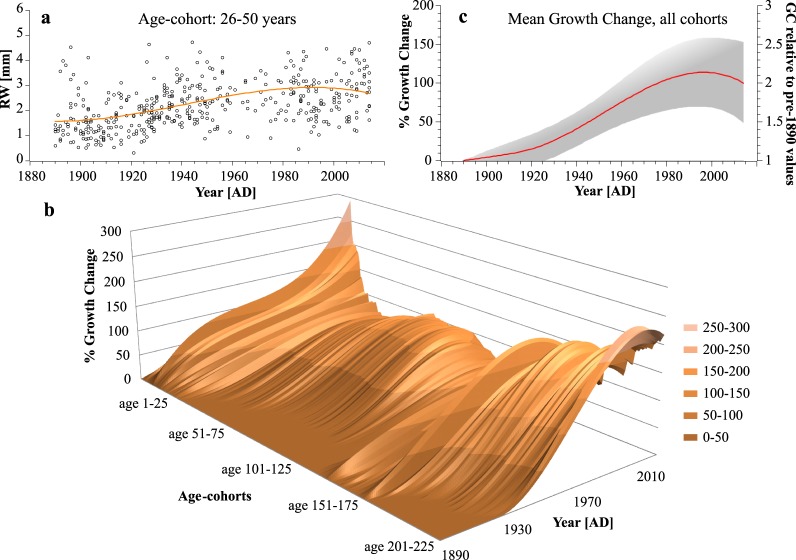
Growth trend detection. (**a**) Example of TRW changes over the last century for the cohort of age 26–50 yrs. and fitted polynomial trend (every dot in the scatterplot represents the mean RW of one individual between 26 and 50 years of cambial age); (**b**) Trend-lines like in a) for all age-cohorts from age 1–25 until age 201–225, shifted one year and plotted with respect to their average TRW in 1890 and (**c**) Mean trend (relative to pre-1890 growth rates) from all age cohorts in b) with standard deviation (grey shading); this curve (right axis) was used to pre-detrend the raw TRW dataset in order to remove human induced non-climatic (fertilization) trends.

**Figure 3:**
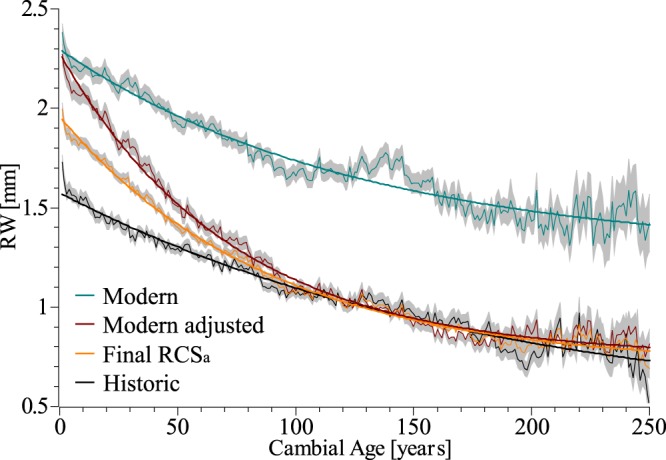
Regional curves of the historic and modern periods. Averages of cambial age aligned TRW-data with standard error (grey shading) for the modern (1700–2014 AD, cyan) and historic (980–1300 AD, black) subsets, the adapted (detrended for the fertilization effect) curve of the modern subset (red) and the final regional curve used for detrending of the whole dataset (RCS_a_, orange). Modern (cyan) and historic (black) curves show a very similar shape which is unusual, as better growing conditions of whatever nature (climate, nutrients, light) should appear as a multiplicative effect in a growth curve and not simply shift the mean (see for example yield tables in forestry). The adjusted curves have a more plausible, scaled shape compared to the historic RC. Mean TRW is 57% higher in the modern part of the dataset (2.00 mm versus 1.27 mm).

**Figure 4:**
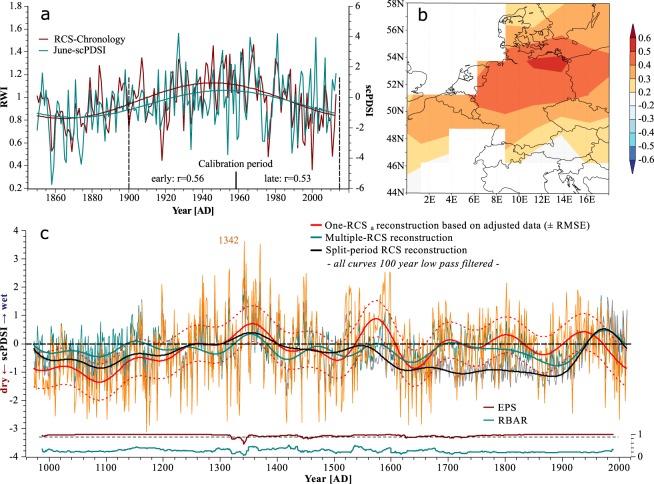
Reconstruction of June scPDSI and fit with instrumental data. (**a**) RCS_a_ chronology (red) together with June scPDSI (cyan). Due to potential inhomogeneity of the climate data, the period from 1901–2014 AD was used for calibration of the final reconstruction; (**b**) spatial field correlations of the reconstruction with gridded data of scPDSI^44^ and (**c**) final reconstruction extending back to 980 AD (red) compared with two alternative reconstructions based on the same dataset but using different detrending methods: split RCS-detrending (black) and multiple, growth rate dependent RCS-detrending (cyan) together with 100 yr. low pass filter (thick lines) and root mean square error (RMSE) of the regression over the calibration period (dashed lines).

Modelling approaches on fertilization effects^29^ and results from fertilizer experiments^30,31^ are still not sufficient to fully explain the observed 20^th^ century increase in tree growth rates^5–8^ that is apparent also in our dataset (Fig. 2). Particularly, the impact of rising CO_2_ concentrations on growth rates and its interplay with other growth relevant factors remains ambiguous as demonstrated by the FACE experiments^32^. Nitrogen on the other hand is assumed to be the limiting nutrient in most forest ecosystems worldwide and atmospheric N-depositions clearly increase growth and carbon sequestration in temperate and boreal forests^6,31,33^, whereas a large scale detrimental effect of pollutants like Sulphur compounds and acid deposition is less pronounced^9,33^. Apparently, interactions between individual driving forces like climate, different atmospheric depositions or land use changes complicate a precise attribution of increasing growth trends to these causes^33–35^. Accordingly, we rejected a mechanistic model and instead developed a data driven approach to remove that part of the long-term growth trend in TRW-data which seems to be related to the no-analogue situation of the recent decades. Already in 1989 a pollution signal termed “P” was added by Fritts & Swetnam^36^ to the linear aggregate model of ring-width measurement, originally introduced by Cook^37^. This pollution term which can also be interpreted as a fertilization signal is assumed to be common to all RW-series of the sampled trees from a region and can be distinguished from climate because of its higher persistence^36^. Therefore an age class isolation method^38,39^ or age-banding^40,41^ was chosen here to detect persistent calendar year effects on growth levels of the trees in our region unaffected by the typical decline of TRW with age (see Methods for details). We consistently found a strong positive calendar year effect on growth rates over the last century throughout all age classes peaking in the 1990’s and levelling out in the last decades (Fig. 2). On average, growth rates (TRW) were 50% higher in the period of strong atmospheric deposition 1950–2010 AD compared to 1890–1950 AD. This increase is in accordance to observational data from long-term monitoring plots, which show up to 77% higher volume increment for beech over the same period^5^. In the absence of any regional climatic trend which could explain such a consistently strong growth increase, enhanced growth since 1950 can be primarily attributed to human influences like nitrogen fertilization or management effects (Supplementary Fig. S11). The reduced slope over the last decades might be attributable to emerging nutritional imbalances of the trees as high levels of N-deposition (currently about 20 kg N/ha*yr. in the study region) are not paralleled by increases in phosphorous input which progressively might become limiting to tree-growth^42,43^. Consequently, we removed this non-climatic bias from the raw proxy data prior to calibration of the reconstruction model. We achieved this by dividing each calendar year aligned TRW-series by the mean 20^th^ century growth change curve from Fig. 2c. This pre-detrending reduced the average growth levels of the modern part of the dataset (Fig. 3) and thereby permitted the application of one common RCS to the entire dataset in order to preserve as much of the low frequency variation in the RW-data as possible (Fig. 3). An illustration of the two-step detrending procedure is provided in Supplementary Fig. S6. The calculated index-chronology was regressed against gridded data of a June drought index (scPDSI^44^; r_1850–2014_ = 0.55), tested for temporal stability of the regression and then used to reconstruct early summer drought conditions to 980 AD. Notably, the long term trends and amplitudes of ring-width index (RWI) and scPDSI data over the instrumental period (1850–2014 AD) show excellent agreement (Fig. 4a). Spatial field correlations revealed significant drought correlations over much of northern Germany/northwest Poland (Fig. 4b). For the purpose of comparison with commonly used methods, two different Index chronologies based on traditional detrending options with differing abilities of retaining long-term trends (split-RCS- and multiple, growth rate dependent RCS-detrending) were assembled and served as a basis for comparative reconstructions (Methods).

### Implications for reconstructed amplitudes

**Figure 5:**
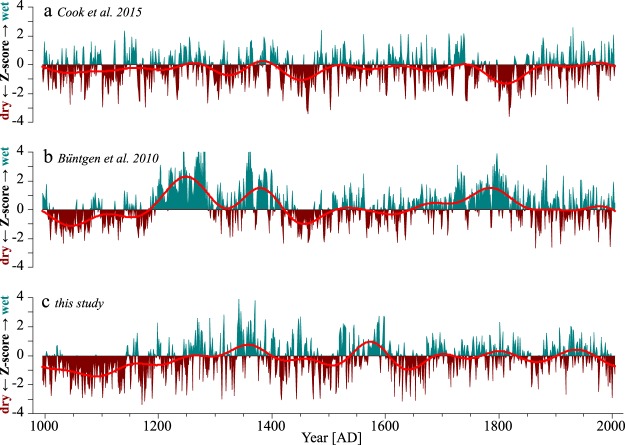
Comparison of RCS_a_ with other tree-ring based drought reconstructions. (**a**) a gridded summer scPDSI reconstruction for the study region based on a multispecies TRW-network^45^, (**b**) a JJAS scPDSI reconstruction for central-west Germany based on oak-TRW^15^, and (**c**) our RCS_a_ early-summer scPDSI reconstruction. Red lines are 100 yr. low pass filters; all reconstructions are z-transformed to have a mean of zero and a standard deviation of one over their whole length and plotted with respect (mean of zero) to the period of instrumental data (1901–2012 AD).

Compared to the traditional reconstruction methods, our approach with the adapted dataset (RCS_a_) retained considerably higher low-frequency variation and notably the MCA was reconstructed as significantly drier (Fig. 4c). The latter holds true when compared to two other tree-ring based summer drought reconstructions for the same^45^ or adjacent areas^15^ (Fig. 5), both derived by applying a split RCS-detrending to the raw TRW-data to circumvent the differing contemporaneous growth-rate bias. A higher negative amplitude during the MCA in our reconstruction is even more noteworthy as in contrast to our conservative linear regression path; Büntgen *et al*. applied a scaling approach in their scPDSI reconstruction (Fig. 5b) which inflates the amplitudes. Significant deviations between the three reconstructions are obvious in the 13^th^ century and around 1800 AD, with two prolonged wet periods in the Büntgen *et al*. data, that are not reproduced by the other two models. This may be partially due to regional climatic differences between central-west (Büntgen *et al*.) and north-east Germany (Cook *et al*. and RCS_a_), but could also originate from differences in proxy behavior (beech/multispecies vs. oak, site-level influences) or in replication (sample depth).

In general, our reconstruction expresses a tendency for drier conditions in the preindustrial past. Notably the period from 1000–1200 AD was reconstructed as persistently dry in the study region with on average ~1 SD lower scPDSI values compared to the 20^th^ century average. This supports previous findings that indicate north-central Europe experienced very pronounced medieval drought^45,46^ but contradicts reconstructions from southern Sweden or the Alps showing sustained wet summers during the same period^47,48^. Importantly, our method facilitates for the first time a direct, hydroclimatic comparison of the MOWP with the MCA. We show that generally warmer temperatures during MCA^49,50^ were accompanied by significantly lower summer precipitation compared with today in the study region. As a parameter that integrates moisture supply (precipitation), demand (potential evapotranspiration) and soil moisture, the scPDSI does not allow for direct inferences of total precipitation amounts. Nevertheless, during the last century only 10–30% of its variability was determined by temperature variations^44^, therefore, summer precipitation must have been significantly reduced to achieve the sustained negative scPDSI values during the MCA in our reconstruction. Simulations with data assimilation have shown that in summer a decreased influence of westerlies and sustained high pressures over Northern Europe which imply reduced cloudiness probably contributed to the warmer and dryer conditions during the MCA^50^. Reconstructed scPDSI values show a good agreement (r = 0.56) with a current reconstruction of the North Atlantic Oscillation (NAO)^51^ which underlines the influence of this large scale circulation pattern on decadal to centennial variations of moisture conditions in the study region (Supplementary Fig. S14). The wettest summer in the RCS_a_ reconstruction was in 1342 AD, which documentary sources record to be among the wettest summers in the last millennium in Central Europe^52^. This nicely shows that our reconstruction is not biased by a greater sensitivity to dry conditions reflected by reduced tree growth, but is also capable of capturing moist years reflecting higher than average growth.

## Conclusion

To summarize, the pronounced differences between our novel RCS_a_ reconstruction and results using more traditional approaches reveal that considerable bias is added to reconstructions which do not account for the current no-analogue situation. Neglecting fertilization effects and other non-climatic anthropogenic influences on tree-growth rates will 1) diminish true long-term trends in reconstructions that are derived by using a differing statistical treatment for the modern and historical/subfossil data (split-detrending); or, 2) lead to lower absolute values of temperature, precipitation, or drought in the pre-instrumental period in case a biased common RC (positively influenced by the modern accelerated tree-growth rates) is applied to uniformly standardize modern and historic/subfossil datasets. This bias will be most pronounced in climate reconstructions for the temperate areas of Europe and North America, which are heavily influenced by novel atmospheric depositions. Nevertheless a fertilization effect cannot be disregarded in the northern, nitrogen limited boreal and high-mountain environments^31,53^, which are the cradles of temperature sensitive tree-ring chronologies widely used for millennial long climate reconstructions^54^. It remains to be tested in these biomes if a positive growth trend attributable to fertilization can be disentangled from the modern warming effect and if MXD, a proxy of late summer temperatures often preferred over TRW because of its higher temperature sensitivity, is similarly affected.

## Methods and Materials

### Tree-ring data

Annually resolved tree-ring width measurements (TRW) from 1896 series of common beech (*Fagus sylvatica* L.) covering the period 959–2014 AD (1055 yrs.) from NE-Germany, a region roughly defined by the Baltic Sea in the North, the Polish border in the East, Berlin in the South and the river Elbe in the West (52.5°–54.5°N; 10.5°–14.5°E) were compiled to form the basis of our analyses (Fig. 1). We selected tree-ring series that passed common quality checks and showed Pearson correlation coefficients higher than 0.3 with the master chronology. The historical part of the dataset entirely consists of series collected by the German Archaeological Institute in Berlin. Intense renovation activities in this region especially during the last decades after the fall of the Berlin wall provided historical beech timber. Many samples originate from the hanseatic-cities: Greifswald, Stralsund, Rostock and Wismar, but wood from smaller inland towns also contributed to the collection. Although beech was only sparsely used as construction wood, radial split planks in old frame houses and logs used in foundations were periodically discovered and dendrochronologically dated. Due to a decrease in construction activities in the region after ~1350 AD^28^ and/or the less frequent use of beech wood in constructions, the replication decreases significantly after this date (see Fig. 1). The high replication of the modern part of our dataset is similar to the early historical dataset and compiled from living tree material growing in closed canopy mature forests distributed over the same region. The 30 sites are representative of diverse local site conditions from poor to nutrient rich to dry, sandy sites over mesic to loamy sites with stagnating wetness. Sampling mainly followed the traditional dendrochronological protocol targeting climate sensitive, dominant trees experiencing little competition. Generally two cores per tree were extracted and measured.

### Disturbance analysis

Preliminary analyses of the modern tree-ring data indicated a strong influence of intense cuttings in the period during and after World War II on TRW patterns of the remaining trees at many sites. The island of Vilm is a rare and strictly protected site now and during the last centuries that hosts an unmanaged beech forest and serves as an undisturbed forest reference. With this reference we could attribute growth releases in the 1940’s and 1950’s elsewhere to World War II driven human influences (Supplementary Fig. S1). In order to remove the non-climatic signal from the TRW-pattern we conducted a disturbance analysis using five year averages of TRW with the boundary line method^55^ and excluded all series crossing the identified boundary line of “normal” growth changes during this period (i.e. those showing growth patterns resembling a release). This decreased the sample size in the modern, living tree part of our dataset from 973 to 783 series with slight implications for the decadal trends (Supplementary Fig. S1). A similar disturbance analysis was conducted for the historic data but apart from scattered single tree releases, no clustered release period could be identified (data not shown). The disturbance analysis was conducted using the TRADER-package^56^ in the R-statistical environment^57^.

### Statistical properties of the ring-width data

The final dataset was split in three periods for comparison of statistical properties: the two highly replicated periods 980–1300 AD (historic) and 1700–2014 AD (modern) and an intermediate period with lower replication (1300–1700 AD). Typical dendro-statistics^58^ were calculated for each subset (Supplementary Table S1), compared and tested for differences (Anova, Tukeys-post hoc test; Supplementary Fig. S2). The main focus was set on the modern-historic subset comparison. The two subsets are similar in their age structure and there are no significant differences in the mean segment length (MSL), however, the historical data is slightly skewed towards younger wood (Fig. 1, Supplementary Fig. S2). The most conspicuous difference is the significantly lower average growth in the historic period. This lower growth is accompanied by higher mean sensitivity (MS), lower first order autocorrelation (AC1^st^) and a higher mean interseries correlation (IC; Supplementary Table S1, Fig. S2). These statistics are all indicative of a higher climate sensitivity or stronger climatic limitations of growth, i.e. the stronger a climatic factor is limiting tree-growth, the higher the interannual TRW-fluctuations (MS) and the common signal (IC) in TRW-patterns of different trees.

### Growth trend analysis and data adaptation

To test for calendar year effects on growth trends we used a cohort- or age class isolation approach^38,39^, also called age-banding^40,41^. This includes the selection of data from certain age class ranges for chronology calculations to eliminate the age effect of decreasing RW. For this purpose the modern subset of the raw TRW-data was aligned by cambial age and averaged in age classes over 25-years starting from age 1–25, progressing in one year steps up to age 201–225, where still a reasonable sample replication (>50 trees) was reached. The samples were then re-arranged to the calendar year of the first ring, and plotted with their mean growth for each respective age class (for an example see Fig. 2a). This age-banding allowed for the detection of calendar year effects (trends) on mean growth rates without age biases. For the modern part of the dataset we detected a strong positive growth trend over the last century in all age-classes. The same analysis applied to the historic subset yielded no consistent trend. In the next step a second order polynomial was individually fitted to each rearranged age cohort and expressed as percentage growth increase relative to the pre-industrial 1890 value (Fig. 2a,b). The resulting 225 curves of long term growth change for the different age cohorts were finally averaged to obtain a mean trend curve over the period of industrialization (1890–2014; Fig. 2c). We eventually used this mean growth increase (absolute values, no percentages, Fig. 2c, right axis) to pre-detrend the raw, modern dataset by dividing the individual series’ raw TRW-values by the mean trend curve (Supplementary Fig. S6a). This adapted dataset formed the basis for chronology computation.

### Artificial data-set

To test how well our cohort method captures a known growth increase and to see if the proposed pre-detrending sufficiently removes this trend, we built an artificial TRW-dataset with known trends on all relevant frequency domains. For this purpose, a white noise, high-frequency time series was simulated and scaled with an idealized age trend following a negative exponential form. Decadal scale variability was added as a sine wave and finally a linear growth trend was multiplied, mimicking the increasing growth trend (fertilization effect) from 1890 AD onwards. The latter two trends were introduced as calendar-year effects. An example of a resulting time series is shown in Supplementary Fig. S3. Starting in 1790 AD we generated 19 series of different age classes from 40 to 220 year length. This dataset was resampled in regular intervals every 20 years (1810, 1830, 1850, … until 1970 AD), resulting in an artificial TRW-dataset of in total 80 pseudo trees (Supplementary Fig. S4). By applying our aforementioned cohort method and a pre-detrending to the raw data before regional curve standardization (RCS), we successfully removed the linear trend over the last century while preserving the desired decadal-scale variability, whereas a traditional regional curve standardization still includes the linear growth increase in the resulting index-chronology (Supplementary Fig. S5). This supports the qualification of our method as appropriate for capturing and removing potential non-climatic growth trends while preserving decadal scale variability.

### Chronology computation

RCS-detrending^19,22^ was used to remove the age trends while attempting to retain as much of the low frequency signal as possible. One common RCS was applied to the entire adjusted (pre-detrended) dataset which allows for direct comparison of the modern with the historic period (Fig. 3). A signal free approach^59,60^ was chosen to additionally reduce potential calendar year effects on the shape of the RC which was smoothed by an age dependent spline curve. Raw values were divided by the smoothed regional curve and their variance was stabilized using the Rbar stabilization method^61^. An example of how the two-step detrending was applied is provided for one example tree in Supplementary Fig. S6. Eventually, a robust (weighted) mean was used to average the indices into the final RCS_a_ chronology. Mean Rbar of the chronology equalled 0.3. The running expressed population signal (EPS) was stable in time and close to one over the whole chronology length with the exception of one period in the middle of the 14^th^ century, where the common signal decreased for some decades during a period of high TRW-amplitudes and rather low sample depth (Fig. 4c). In general, these metrics indicate that the population signal is well represented by our chronology^62,63^. In addition to the one-curve RCS-chronology based on the adjusted dataset (RCS_a_), we calculated two alternative chronologies based on the original data for the purpose of comparing our new-method to more traditional procedures. First, for the split-detrending approach, separate regional curves were fitted to the modern, intermediate and historic sub periods and second, in the multiple RCS-approach, we fitted five different growth-rate dependent regional curves to the dataset. The potential to retain low-frequency information in the final chronologies decreases with increasing flexibility of the detrending curves- from the one common RCS_a_-detrending over the split period- to the growth-rate dependant RCS-detrending^21,64^.

Again a pseudo-tree exercise with known trends an all frequency domains was carried out to show that even if our common-RC_a_ is above the mean growth of many historic series it correctly reproduces the original long-term centennial trends (Supplementary Figs S7–S10). A prerequisite for not biasing the shape and mean of the common RC to one of the parts of the dataset is a similar sample replication in the modern and historic part, which is met by our dataset. Split detrending on the other hand is not able to retain trends on frequencies longer than the period used for deriving the two (or more) regional curves (Supplementary Fig. S10).

### Climate of the study region

The sample area is predominantly influenced by western circulations (especially in winter), but the Atlantic influence decreases gradually towards the east^65^. Mean annual temperature in the 20^th^ century averaged over 10.5–14.5°E and 53.0–54.5°N, was 8.6 °C; annual precipitation totals were 610 mm with about 55% falling during the growing season (April-September). Towards the eastern part of the study area beech already approaches its distribution limit with annual precipitation totals decreasing to around 500 mm. Over the past century, there was a significant trend of increasing temperature of about 1 °C, most pronounced in the decades after 1990, whereas annual precipitation and drought indices fluctuated without significant long term trends (Supplementary Fig. S11).

### Calibration and reconstruction

Previous studies^26,27^ and preliminary analyses revealed a strong and temporally stable drought signal in beech-TRW in the study area, as expressed by positive correlations with summer precipitation and co-occurring negative correlations with summer temperature. Drought indices like the Standardized Precipitation Evapotranspiration Index (SPEI) or the self-calibrating Palmer Drought Severity Index (scPDSI) which combine the aforementioned factors show the highest correlations with beech TRW. A gridded version of monthly scPDSI available from 1850–2014 AD^44^ and averaged over the study area (10.5°–14.5°E; 52.5°–54.5°N) was used here as predictor variable in growth response analysis. The strongest correlation was obtained with June values which - due to the highly autocorrelative structure of scPDSI-calculation- are indicative of early summer drought conditions. We therefore abstained from averaging several months to seasonal values and used the June scPDSI instead. Moving window correlations between our chronology and June scPDSI were significant over the whole period of overlap, but fluctuated between r = 0.34 and r = 0.77 with periods of weaker correlations at the end of the 19^th^/beginning of the 20^th^ century and also in recent years (Supplementary Fig. S12). The Pearson correlation coefficient over the common period (1851–2014 AD) was 0.55.

Using ordinary least square regression (OLSR) with the chronologies as independent and June scPDSI as dependent variable, we transferred the three different index chronologies (RCS_a_ and two traditional) into early summer drought reconstructions back to 973 AD. Compared to scaling (i.e. the simple adjustment of the mean and variance of the chronology to the values of the climate parameter), OLSR provides conservative estimates. In OLSR the variability and amplitudes of the reconstructions are reduced by the unexplained variance^66^ and therefore the reconstructed climate is biased towards the mean. Nevertheless OLSR provides robust error estimates whereas scaling inevitably results in increasing errors and a loss in reconstruction skill. It has been demonstrated^67^ that the equivalent variance explained (R²_vs_) of a scaling approach can be calculated with the formula (1) R²_vs_ = 2 │r│ −1 using simply the Pearson correlation coefficient (r). Applying this formula to our data would reduce the explained variance from an R² of 30% (linear regression) to an R²_vs_ of only 10% (scaling), which we perceived as insufficient.

To assess the temporal stability of the linear models, cross calibration/verification was applied using 50% of the 165 years of overlap between instrumental data and the chronologies. We calculated reduction of error (RE), Pearson correlations (r), root mean square error (RMSE) and coefficient of efficiency (CE) statistics for all periods together with Durban Watson (DW) statistics for estimation of autocorrelation in the model-residuals (Supplementary Table S2). It turned out that the model residuals were significantly autocorrelated over the early calibration period (1850–1931) for all chronologies, as indicated by DW-values ≤1.5. This might be due to a deterioration of climate data quality before 1900 AD (temporal inhomogeneity)^68^; we therefore decided to use the period 1901–2014 with more robust climate data for calibration/verification trials. The final RCS_a_ chronology passed all stability tests with positive RE- and CE-values and insignificant DW-statistics, whereas the split-RCS and multi-RCS chronologies displayed temporal instabilities as expressed by negative RE and CE statistics in the late calibration period (1958–2014 AD) (Supplementary Table S2). Our final reconstructions were build using the 1901–2014 AD period for calibration. Differences between the four reconstructions based on different detrending methods are shown in Supplementary Fig. S13 for the two highly replicated periods 980–1300 AD and 1700–2014 AD, and for the whole period in Fig. 4 of the main text.

## Supplementary information

Supplementary Information

Dataset 1

## Data Availability

Data supporting the findings of this study are available in this article and its Supplementary Information files, or from the corresponding author upon request.
