# Supplementary material for: Removing the no-analogue bias in modern accelerated tree growth leads to stronger medieval drought
Source: Sci Rep. 2019 Feb 21;9:2509. doi: 10.1038/s41598-019-39040-5 (PMC6385214; doi:10.1038/s41598-019-39040-5)
Supplement: Supplementary file 1 — Supplementary Information [file 41598_2019_39040_MOESM1_ESM.pdf]

Supplementary Material for ‘Removing the no-analogue bias in modern accelerated tree growth leads to stronger medieval drought’

Tobias Scharnweber, Karl-Uwe Heußner, Marko Smiljanic, Ingo Heinrich, Marieke van der Maaten-Theunissen, Ernst van der Maaten, Thomas Struwe, Allan Buras & Martin Wilmking

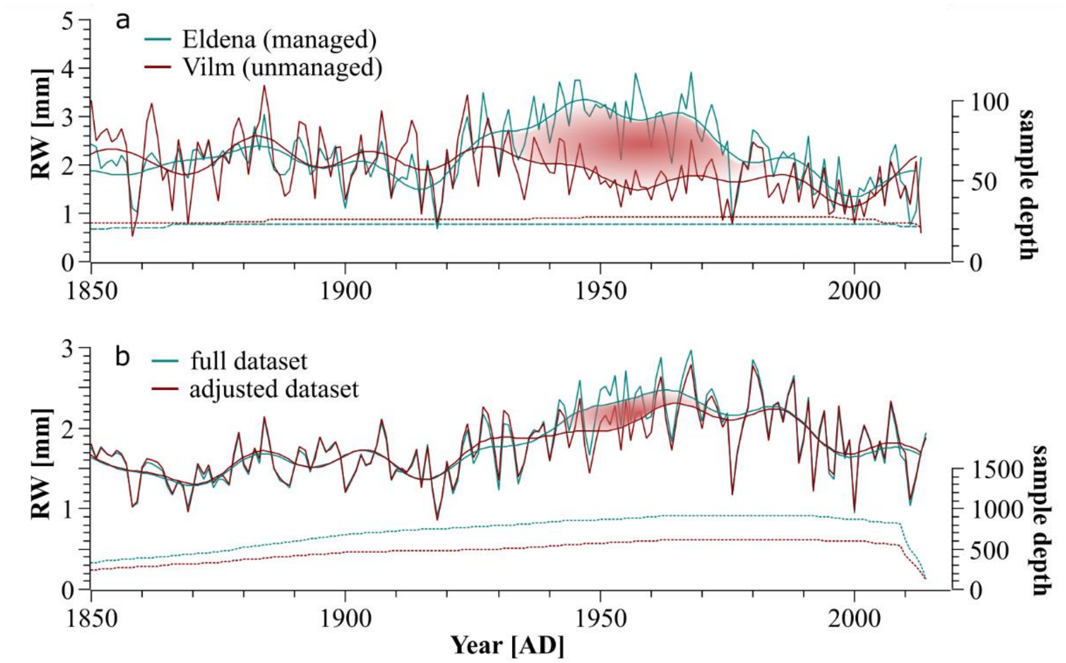

Figure S1: **a**, Raw chronologies (simple TRW-averages) of two, in terms of stand age, tree density and nutrient status comparable beech-forests in short geographical distance to each other. Vilm has no forestry history for >300 years and shows no growth release around the 1940´s (red shaded area) whereas Eldena clearly shows a growth release in this period which can be attributed to documented heavy cuttings related to World War II, and **b**, Raw chronologies of the original dataset used in this study before and after adjustment for release effects

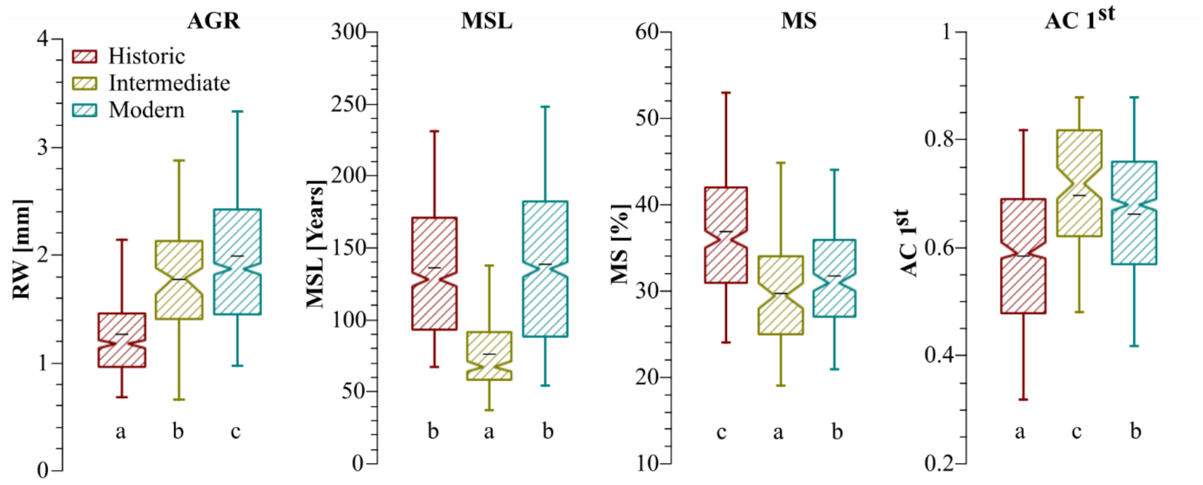

Figure S2: Comparison of statistical properties between the three chronology periods. Differences are tested with a one-way Anova and Tukey´s post hoc test (small letters; p<0.05)); AGR: average growth rate [mm]; MSL: mean segment length [years]; MS: mean sensitivity [%]; AC 1st : 1st order autocorrelation

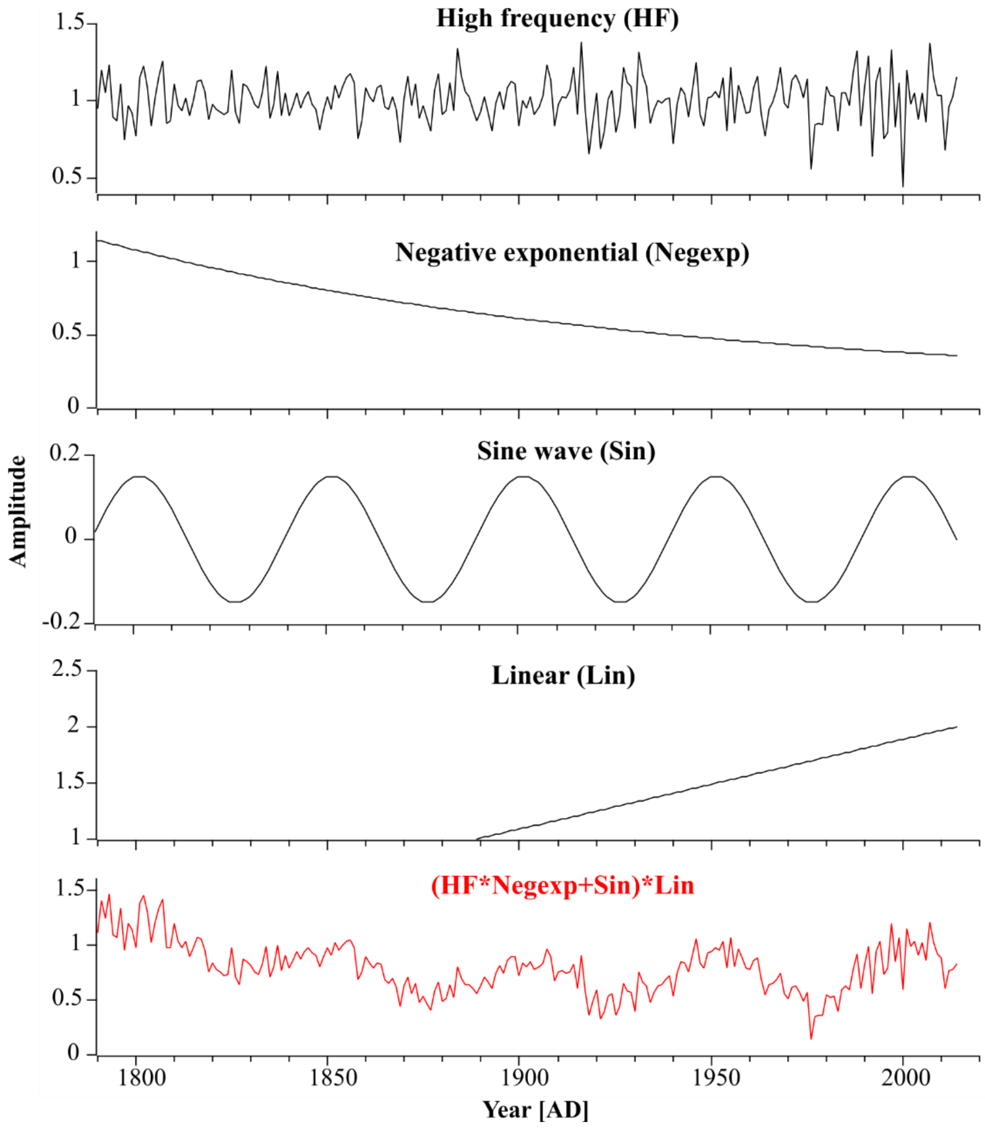

Figure S3: Artificial time series example: a high frequency signal is scaled with a negative exponential curve (modelled as cambial age effect) and decadal scale variability is added in form of a sine wave, the resulting time series is finally scaled with a linear trend starting in 1890 (both modelled as calendar year effects)

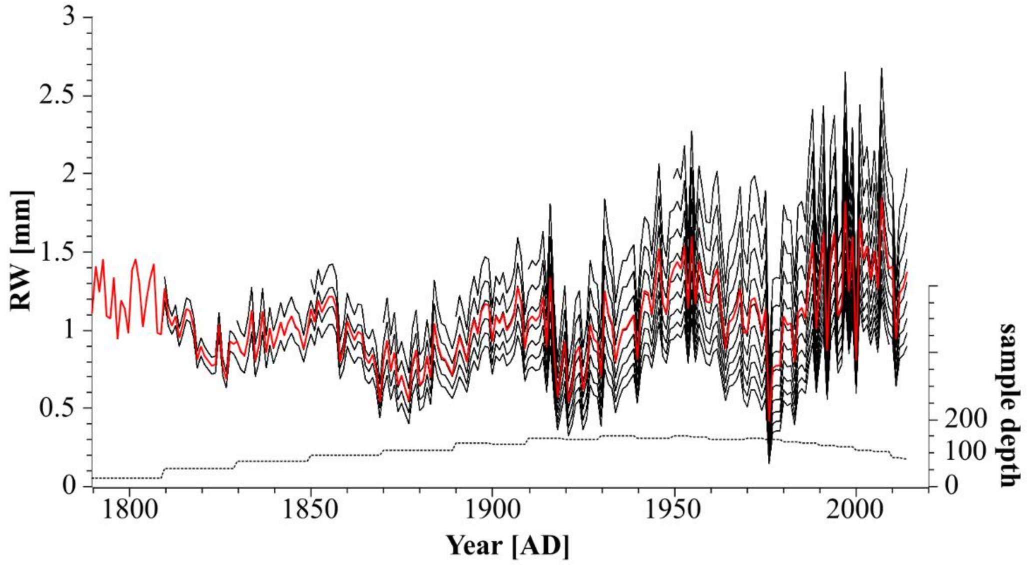

Figure S4: Raw artificial dataset: time series of different length (age) are constructed like in the example of Fig. S3 and resampled every 20 years; the red line is the raw average of all 80 series

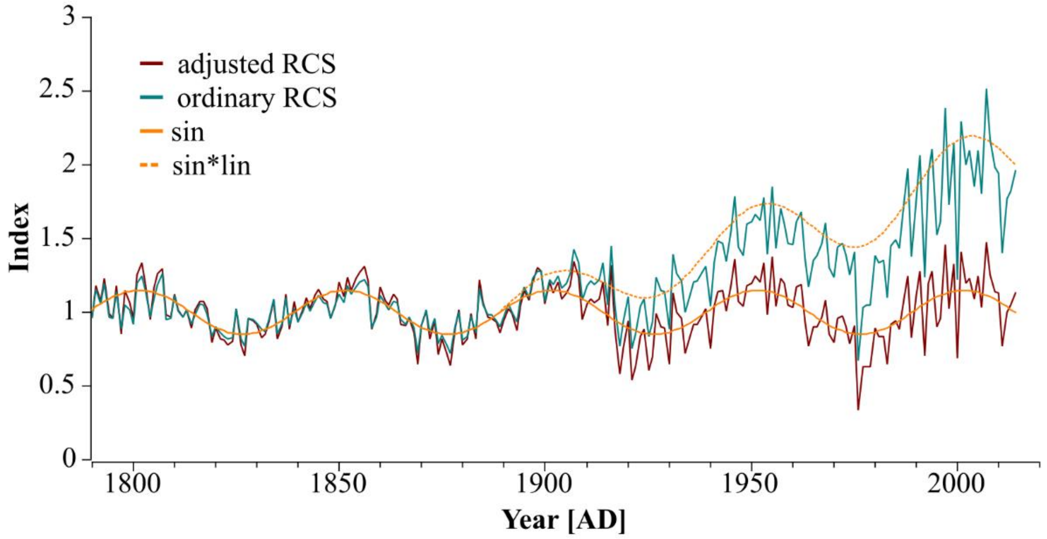

Figure S5: Chronologies of differently detrended series based on the artificial dataset from Fig. S4; the ordinary RCS (cyan curve) still includes the linear trend from 1890 on, whereas the RCS of the pre-detrended (adjusted) dataset using the cohort method correctly removed the linear increase (red curve); the underlying sine-curves from the initial artificial dataset (see Fig. S3) are plotted in orange for comparison

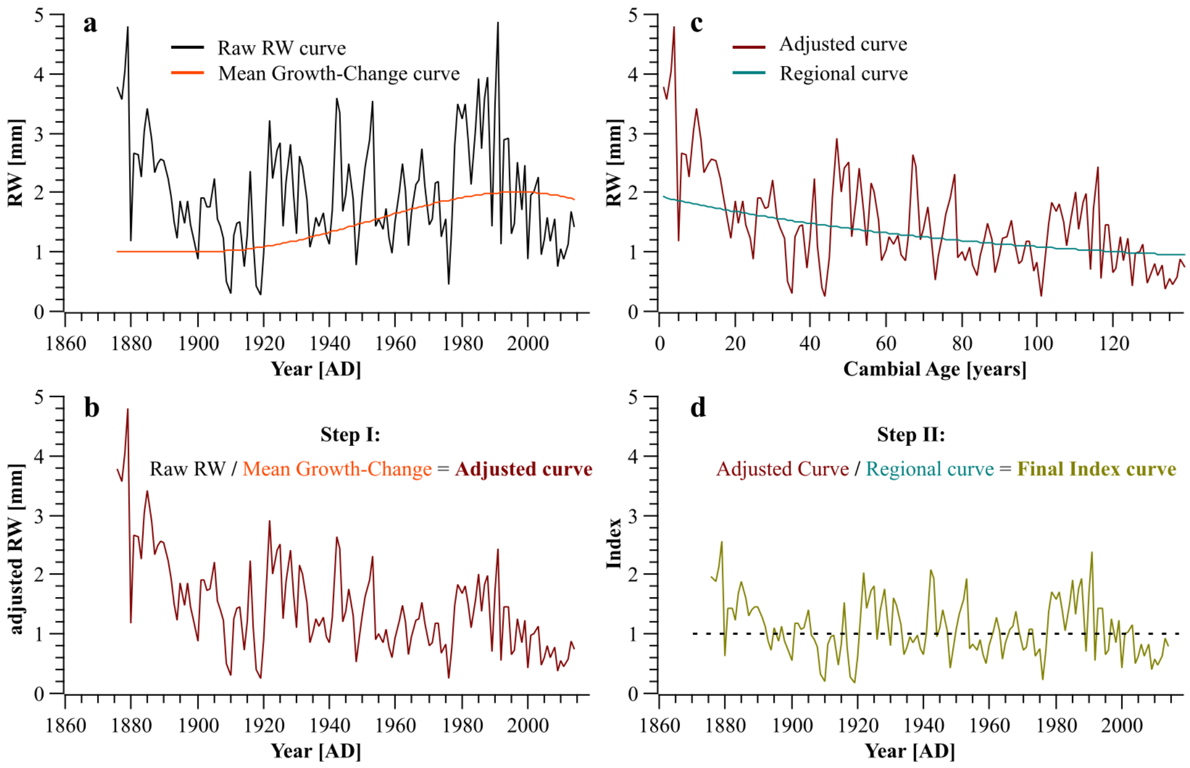

Figure S6: Example of how the two-step detrending for chronology computation was carried out: First the raw RW-measurements were divided by the mean growth change curve (see Fig.2 in the main manuscript): panels **a & b**. The resulting adjusted RW-curve was in a second step divided by the regional curve derived from the complete dataset (orange curve in Fig.3 main manuscript): panels **c & d**.

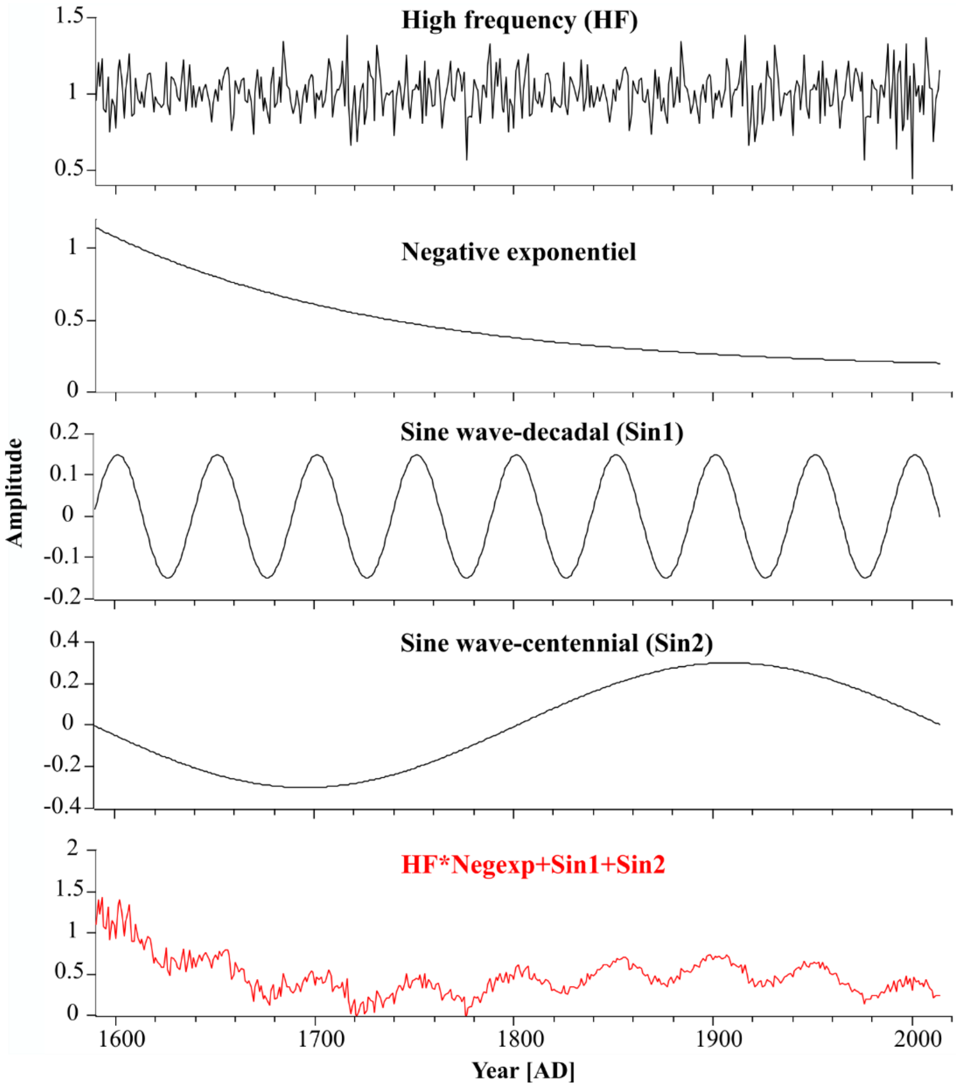

Figure S7: Artificial time series example: a high frequency signal is scaled with a negative exponential curve (modelled as cambial age effect) and decadal and centennial scale variability are added in form of two different sine waves (both modelled as calendar year effects)

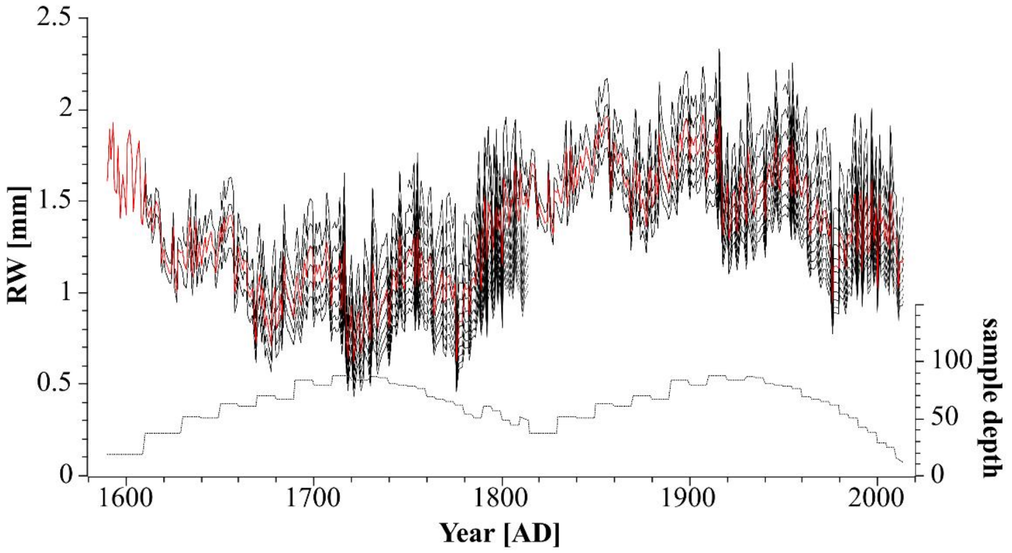

Figure S8: Raw artificial dataset: time series of different length (age) are constructed like in the example of Fig. S6 and resampled every 20 years; the dataset was replicated over an earlier (historic1590-1814 AD) and later (modern) period (1790-2014 AD); the red line is the raw average of all 160 series

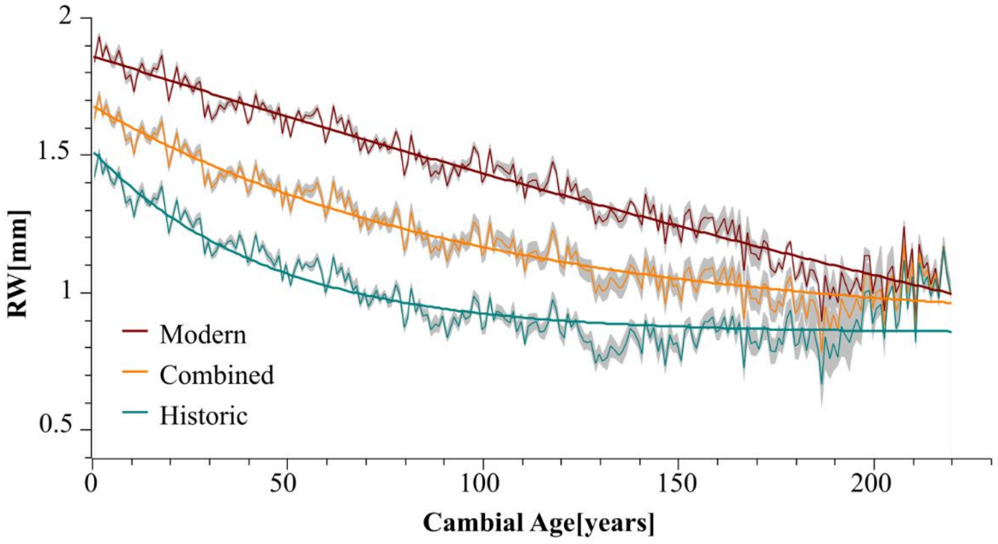

Figure S9: Regional curves (averages of the cambial age aligned RW-series) of the modern (red); historic (cyan) and combined part of the pseudo tree dataset in Fig. S8 with standard error (grey shading)

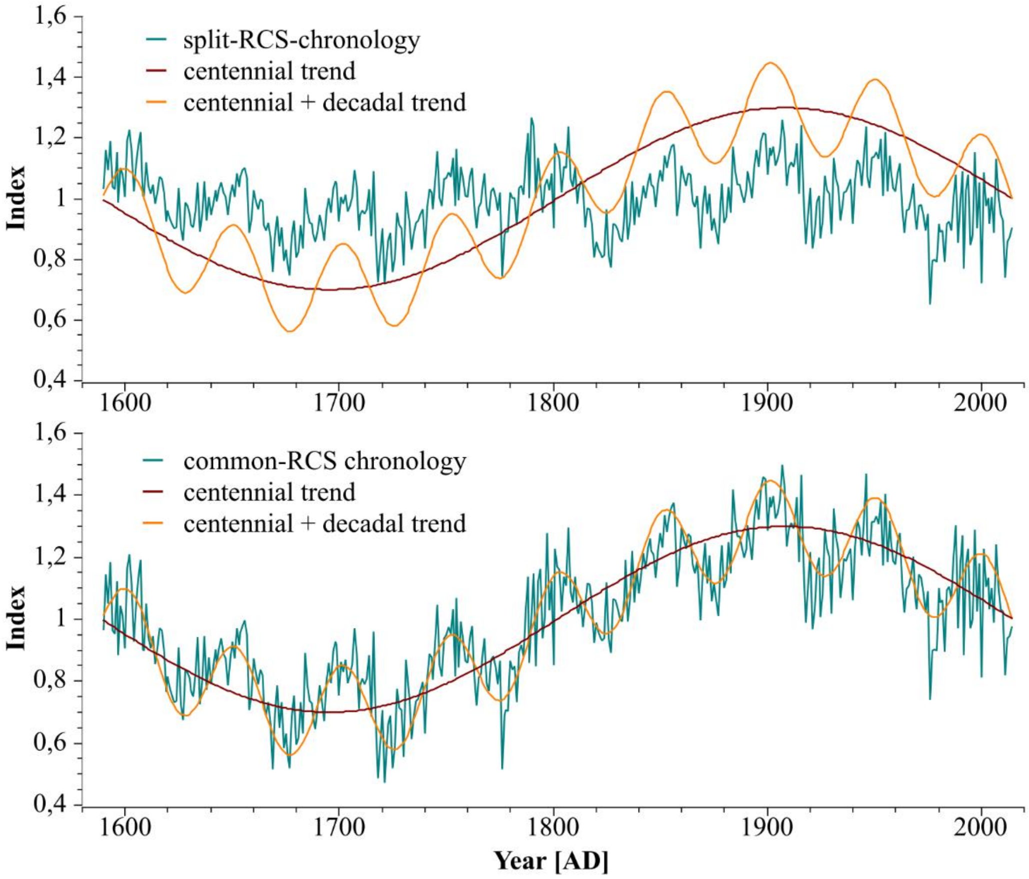

Figure S10: Chronologies of differently detrended series based on the artificial dataset from Fig. S7; the split detrending of the modern and historic parts of the dataset captures the decadal scale variability but the centennial trend is lost (upper panel) whereas the common RCS detrending (lower panel) using a common detrending curve for both parts of the pseudo-tree dataset correctly retains the trends on both frequency domains; the underlying original trends from the artificial dataset (see Fig.S6) are plotted for comparison in orange and red

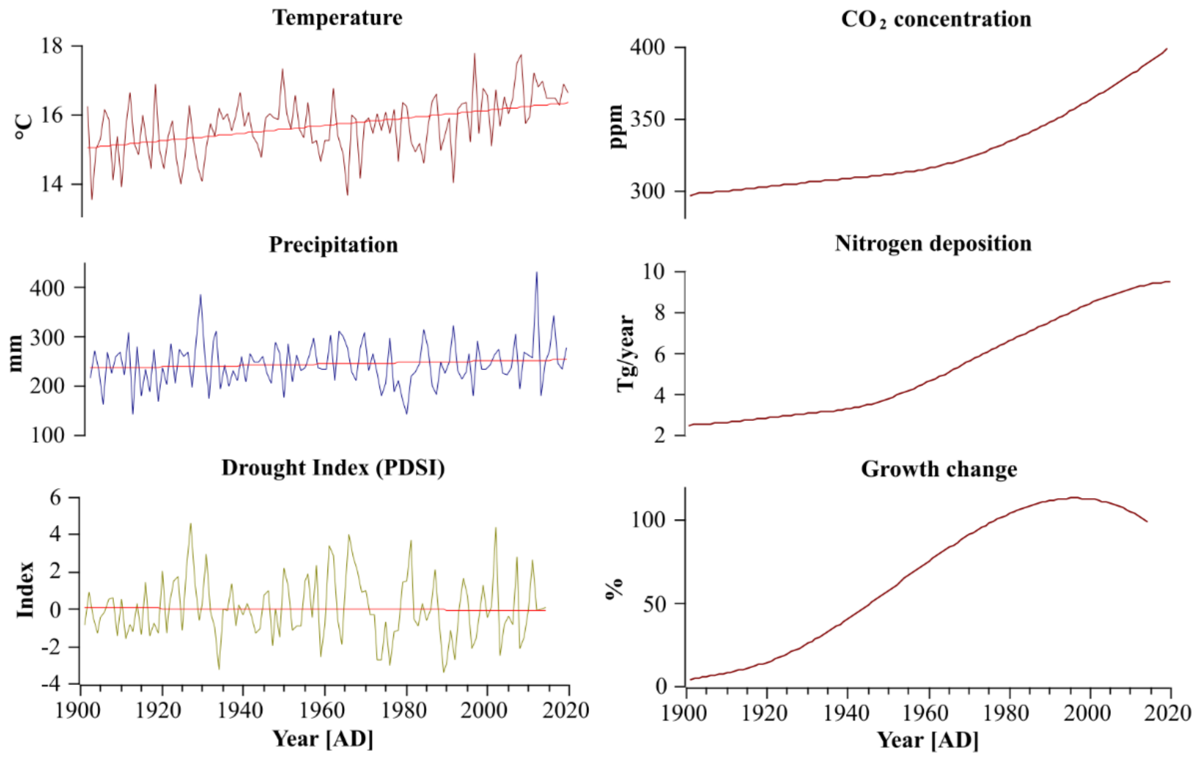

Figure S11: Left: Temperature, precipitation and drought variability over the last 114 years (1901-2015 AD) averaged/summed up for May –August, the main period of ring formation, values are gridded data from CRU-TS 4.0 (T & P)[1] and Dai et al. (2004)[2] (self-calibrated Palmer Drought Severity Index, scPDSI) averaged over the study region (10.5-14.5°E and 53.0-54.5°N) Right: Trends in atmospheric CO_2 concentrations and atmospheric Nitrogen deposition (wet and dry) over Europe from Churkina et al. (2010)[3] together with the percentage growth change of beech from this study

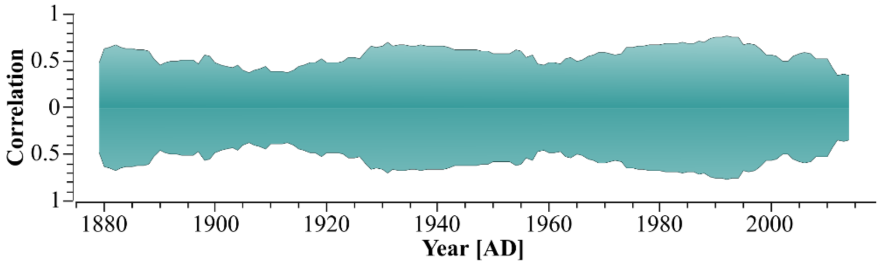

Figure S12: Butterfly plot of moving correlations (30 year windows, last year plotted) between June-scPDSI and the final RCS_a-chronology over the common period 1850-2014 AD

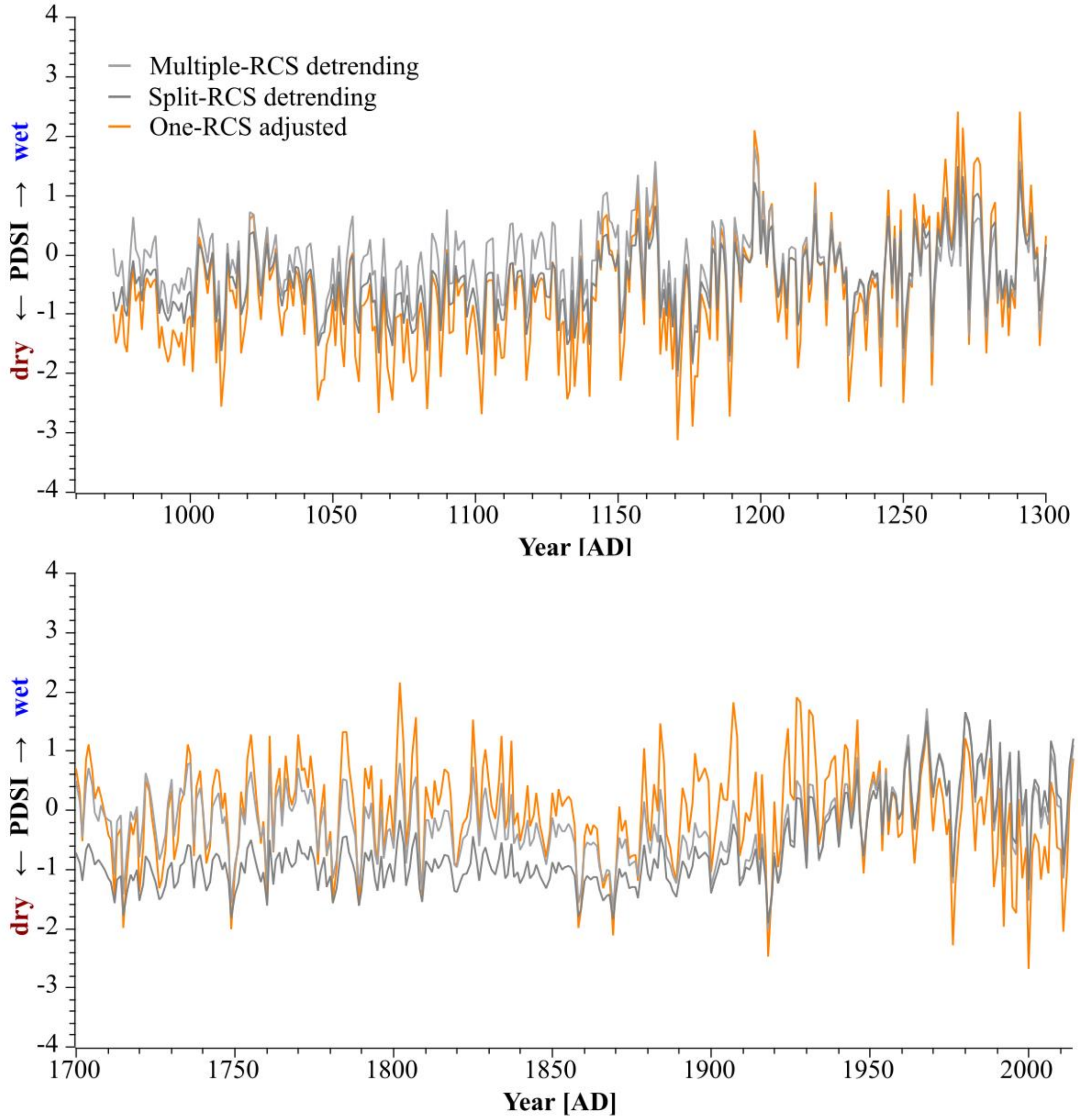

Figure S13: Comparison of three different early summer scPDSI reconstructions based on the same beech dataset but using different detrendings over the highly replicated modern and historic periods; over the modern period (lower panel) reconstructed values of RCSadjusted are comparably wetter whereas over the historic period (upper panel) the other two reconstructions display lesser drought

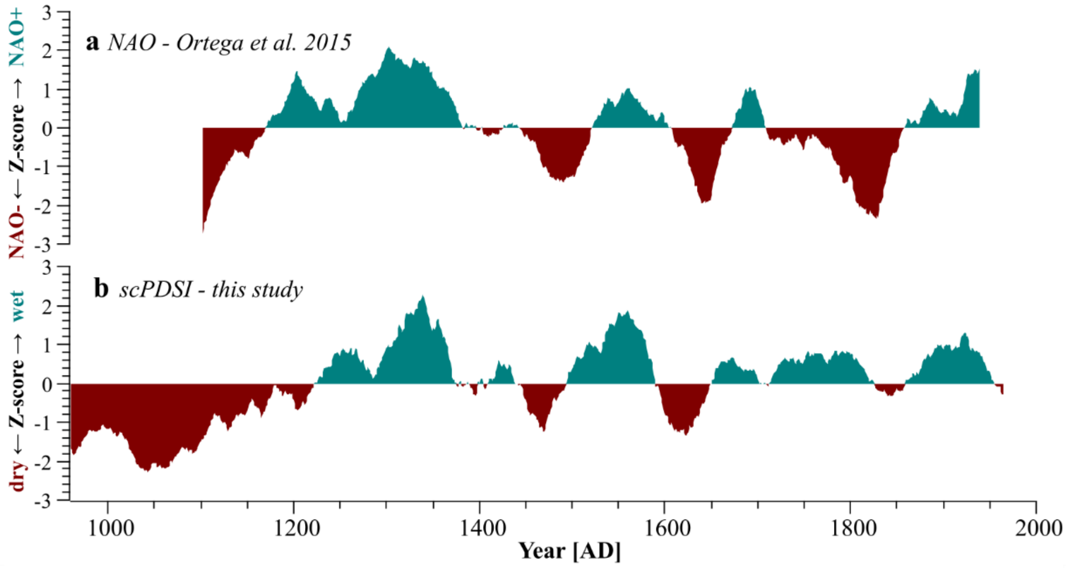

Figure S14: Comparison of our RCSa early-summer scPDSI reconstruction **(b)** with a reconstruction of the North Atlantic Oscillation (NAO)[4]; **(a)**. Both curves are z-transformed and smoothed with a 50-year moving average to emphasize coherence on lower frequencies.

Table S1: Summary of statistical data properties: sample depth, mean segment length (MSL), average growth rate (AGR), mean sensitivity (MS), 1st order autocorrelation (AC 1st) and the mean interseries correlation (IC), all calculated for the three subsets and the complete dataset

Subsets	No of Series	MSL [years]	AGR [mm] ±Std.dev	MS [%]	AC 1st	IC
Modern	676	140	2.00±0.94	33	0.68	0.52
Intermediate	140	78	1.79±0.87	31	0.71	0.56
Historic	783	136	1.27±0.62	37	0.59	0.58
all	1599	132	1.62±0.78	35	0.64	0.55

Table S2: Statistics of the reconstruction models based on different detrending methods; critical values in red. Because of significant autocorrelation in the model residuals over the early calibration period (1850-1931) indicated by Durban-Watson statistics ≤1.5, the final reconstructions were built using the period from 1901-2014 which was split for calibration-verification trials; reduction of error (RE); coefficient of efficiency (CE); root mean square error (RMSE); Pearson correlation coefficient (r); DW-Durban-Watson statistic (DW)

1850-2014 AD

	early calibration/late verification 1850-1931/1932-2014					late calibration/early verification 1932-2014/1850-1931					full model 1850-2014
	RE	CE	RMSE	r	DW	RE	CE	RMSE	r	DW	r (R^2)
RCS	0.21	0.07	0.76	0.62	1.49	0.36	0.22	0.72	0.46	1.72	0.55 (0.30)
SplitRCS	-1.25	-1.64	2.5	0.67	1.56	0.17	-0.01	1.06	0.49	1.8	0.52 (0.27)
MultRCS	0.3	0.18	0.57	0.48	1.29	0.22	0.12	0.59	0.52	1.84	0.52 (0.27)

1901-2014 AD

	early calibration/late verification 1901-1957/1958-2014					late calibration/early verification 1958-2014/1901-1957					full model 1901-2014
	RE	CE	RMSE	r	DW	RE	CE	RMSE	r	DW	r (R^2)
RCS	0.19	0.19	0.64	0.56	1.98	0.13	0.13	0.67	0.53	1.68	0.53 (0.28)
SplitRCS	0.02	0.02	0.98	0.46	1.93	-0.45	-0.45	1.3	0.52	1.66	0.42 (0.18)
MultRCS	0.17	0.16	0.56	0.42	1.97	-0.24	-0.25	0.99	0.53	1.68	0.44 (0.18)

References

1. Harris, I., Jones, P. D., Osborn, T. J. & Lister, D. H. Updated high-resolution grids of monthly climatic observations–the CRU TS3. 10 Dataset. *Int. J. Climatol.* **34,** 623–642 (2014).

2. Dai, A., Trenberth, K. E. & Qian, T. A global dataset of Palmer Drought Severity Index for 1870–2002: relationship with soil moisture and effects of surface warming. *J. Hydrometeorol.* **5,** 1117–1130 (2004).

3. Churkina, G. *et al.* Interactions between nitrogen deposition, land cover conversion, and climate change determine the contemporary carbon balance of Europe. *Biogeosciences* **7,** 2749–2764 (2010).

4. Ortega, P. *et al.* A model-tested North Atlantic Oscillation reconstruction for the past millennium. *Nature* **523,** 71 (2015).
