# Supplementary material for: Removing the no-analogue bias in modern accelerated tree growth leads to stronger medieval drought
Source: Sci Rep. 2019 Feb 21;9:2509. doi: 10.1038/s41598-019-39040-5 (PMC6385214; doi:10.1038/s41598-019-39040-5)
Supplement: Supplementary file 2 — Dataset 1 [file 41598_2019_39040_MOESM2_ESM.docx]

Supplementary Data for ‘Removing the no-analogue bias in modern accelerated tree growth leads to stronger medieval drought’

Tobias Scharnweber, Karl-Uwe Heußner, Marko Smiljanic, Ingo Heinrich, Marieke van der Maaten-Theunissen, Ernst van der Maaten, Thomas Struwe, Allan Buras & Martin Wilmking

|  | **Reconstructions of June scPDSI based on:** | | |
| --- | --- | --- | --- |
| **Year [AD]** | **1RCS-adjusted detrending** | **Multiple-RCS detrending** | **Split-period RCS detrending** |
| 973 | -1.0079998 | 0.083733355 | -0.640668504 |
| 974 | -1.50238204 | -0.3224726 | -0.955081012 |
| 975 | -1.34736388 | -0.36923012 | -0.849494722 |
| 976 | -0.84460228 | -0.10037438 | -0.525696766 |
| 977 | -1.48562332 | -0.652697585 | -0.931617392 |
| 978 | -1.6364518 | -0.863106425 | -1.027818234 |
| 979 | -0.59322148 | 0.0866557 | -0.370836874 |
| 980 | -0.18682252 | 0.63021187 | -0.09631252 |
| 981 | -0.79013644 | 0.09250039 | -0.488154974 |
| 982 | -0.60579052 | -0.068228585 | -0.370836874 |
| 983 | -1.27194964 | -0.535803785 | -0.786142948 |
| 984 | -0.6099802 | 0.10418977 | -0.370836874 |
| 985 | -0.39630652 | 0.05158756 | -0.244133326 |
| 986 | -0.55970404 | -0.050694515 | -0.337987806 |
| 987 | -0.45077236 | 0.2035495 | -0.269943308 |
| 988 | -0.40468588 | 0.31459861 | -0.244133326 |
| 989 | -1.58617564 | -0.839727665 | -0.983237356 |
| 990 | -1.20072508 | -0.430599365 | -0.739215708 |
| 991 | -1.52333044 | -0.489046265 | -0.94100284 |
| 992 | -1.81660804 | -0.991689605 | -1.124019076 |
| 993 | -1.65321052 | -0.804659525 | -1.02312551 |
| 994 | -1.26775996 | -0.506580335 | -0.7767575 |
| 995 | -1.33898452 | -0.4978133 | -0.818992016 |
| 996 | -1.56103756 | -0.65561993 | -0.959773736 |
| 997 | -1.29708772 | -0.389686535 | -0.809606568 |
| 998 | -1.86269452 | -0.600095375 | -1.15217542 |
| 999 | -1.10436244 | -0.214345835 | -0.673517572 |
| 1000 | -1.0289482 | -0.249413975 | -0.626590332 |
| 1001 | -1.98419524 | -0.97123319 | -1.231951728 |
| 1002 | -0.68120476 | -0.11790845 | -0.420110476 |
| 1003 | 0.29918036 | 0.62436718 | 0.18525092 |
| 1004 | 0.13578284 | 0.428570065 | 0.09139644 |
| 1005 | -0.14073604 | 0.224005915 | -0.082234348 |
| 1006 | -0.66025636 | -0.144209555 | -0.417764114 |
| 1007 | -0.28318516 | 0.270763435 | -0.159664294 |
| 1008 | 0.04360988 | 0.36135613 | 0.025698304 |
| 1009 | -1.82917708 | -1.12319513 | -1.14044361 |
| 1010 | -1.07922436 | -0.42183233 | -0.673517572 |
| 1011 | -2.56656076 | -1.617071435 | -1.614408734 |
| 1012 | -1.78728028 | -0.898174565 | -1.114633628 |
| 1013 | 0.05198924 | 0.200627155 | 0.028044666 |
| 1014 | -0.60579052 | -0.313705565 | -0.384915046 |
| 1015 | -0.59741116 | -0.07115093 | -0.361451426 |
| 1016 | -0.9032578 | -0.325394945 | -0.56558492 |
| 1017 | 0.13578284 | 0.25030702 | 0.074971906 |
| 1018 | -1.66158988 | -0.98292257 | -1.02312551 |
| 1019 | -1.2384322 | -0.775436075 | -0.7767575 |
| 1020 | -0.89487844 | -0.46858985 | -0.572624006 |
| 1021 | 0.60083732 | 0.726649255 | 0.344803536 |
| 1022 | 0.67625156 | 0.67696939 | 0.3964235 |
| 1023 | 0.081317 | 0.299986885 | 0.035083752 |
| 1024 | -0.66863572 | -0.068228585 | -0.434188648 |
| 1025 | -1.10436244 | -0.62055179 | -0.69463483 |
| 1026 | -0.18682252 | 0.107112115 | -0.150278846 |
| 1027 | 0.37878428 | 0.475327585 | 0.206368178 |
| 1028 | -0.4214446 | -0.167588315 | -0.288714204 |
| 1029 | -0.23709868 | 0.11003446 | -0.15497157 |
| 1030 | 0.11902412 | 0.352589095 | 0.025698304 |
| 1031 | -0.82365388 | -0.278637425 | -0.537428576 |
| 1032 | -1.37669164 | -0.70822214 | -0.87061198 |
| 1033 | -0.96191332 | -0.424754675 | -0.617204884 |
| 1034 | -0.91163716 | -0.407220605 | -0.586702178 |
| 1035 | -0.37535812 | 0.007752385 | -0.267596946 |
| 1036 | -0.86555068 | -0.29909384 | -0.574970368 |
| 1037 | -0.26223676 | 0.12172384 | -0.206591534 |
| 1038 | -0.35022004 | 0.12756853 | -0.227708792 |
| 1039 | -0.80270548 | -0.2055788 | -0.528043128 |
| 1040 | -1.34736388 | -0.681921035 | -0.854187446 |
| 1041 | -0.47172076 | 0.118801495 | -0.316870548 |
| 1042 | -0.30413356 | 0.200627155 | -0.234747878 |
| 1043 | -0.7775674 | -0.19388942 | -0.511618594 |
| 1044 | -1.44372652 | -0.69653276 | -0.922231944 |
| 1045 | -2.4534394 | -1.424196665 | -1.544017874 |
| 1046 | -2.11407532 | -1.213787825 | -1.325806208 |
| 1047 | -2.10569596 | -1.137806855 | -1.314074398 |
| 1048 | -1.51914076 | -0.75497966 | -0.962120098 |
| 1049 | -1.28870836 | -0.5562602 | -0.814299292 |
| 1050 | -0.73148092 | -0.05946155 | -0.492847698 |
| 1051 | -1.54008916 | -0.74913497 | -0.955081012 |
| 1052 | -0.3795478 | 0.159714325 | -0.279328756 |
| 1053 | -1.48562332 | -0.57963896 | -0.933963754 |
| 1054 | -1.8878326 | -0.892329875 | -1.185024488 |
| 1055 | -0.86974036 | -0.11206376 | -0.593741264 |
| 1056 | -0.07370116 | 0.44902648 | -0.103351606 |
| 1057 | 0.03104084 | 0.66528001 | -0.007150764 |
| 1058 | -1.71186604 | -0.810504215 | -1.067706388 |
| 1059 | -2.13502372 | -1.149496235 | -1.323459846 |
| 1060 | -0.85717132 | -0.150054245 | -0.558545834 |
| 1061 | -0.83622292 | -0.033160445 | -0.521004042 |
| 1062 | -0.73148092 | 0.01651942 | -0.47173044 |
| 1063 | -0.49266916 | 0.264918745 | -0.3074851 |
| 1064 | -1.28870836 | -0.34585136 | -0.81195293 |
| 1065 | -1.1965354 | -0.266948045 | -0.757986604 |
| 1066 | -2.66711308 | -1.593692675 | -1.658989612 |
| 1067 | -1.41858844 | -0.500735645 | -0.896421962 |
| 1068 | -0.58065244 | 0.276608125 | -0.375529598 |
| 1069 | -1.89621196 | -0.89525222 | -1.170946316 |
| 1070 | -2.1392134 | -1.091049335 | -1.32815257 |
| 1071 | -2.44506004 | -1.394973215 | -1.525246978 |
| 1072 | -1.06246564 | -0.237724595 | -0.675863934 |
| 1073 | -1.04151724 | -0.2055788 | -0.666478486 |
| 1074 | -0.98286172 | -0.091607345 | -0.638322142 |
| 1075 | -1.82917708 | -0.80758187 | -1.154521782 |
| 1076 | -1.070845 | -0.179277695 | -0.678210296 |
| 1077 | -1.78728028 | -0.80758187 | -1.11697999 |
| 1078 | -2.13083404 | -1.126117475 | -1.332845294 |
| 1079 | -1.98000556 | -0.968310845 | -1.23429809 |
| 1080 | -1.05827596 | -0.167588315 | -0.68290302 |
| 1081 | -0.79432612 | 0.048665215 | -0.521004042 |
| 1082 | -1.46467492 | -0.576716615 | -0.955081012 |
| 1083 | -2.60426788 | -1.529401085 | -1.619101458 |
| 1084 | -1.71186604 | -0.833882975 | -1.10524818 |
| 1085 | -0.35440972 | 0.36135613 | -0.253518774 |
| 1086 | -0.53037628 | 0.200627155 | -0.347373254 |
| 1087 | -0.88649908 | -0.103296725 | -0.560892196 |
| 1088 | -2.06379916 | -1.09397168 | -1.288264416 |
| 1089 | -0.7985158 | -0.08284031 | -0.504579508 |
| 1090 | 0.09388604 | 0.77048443 | 0.023351942 |
| 1091 | -1.31803612 | -0.489046265 | -0.837762912 |
| 1092 | -1.280329 | -0.471512195 | -0.814299292 |
| 1093 | -0.24547804 | 0.41395834 | -0.187820638 |
| 1094 | -0.68958412 | 0.07496632 | -0.462344992 |
| 1095 | -0.55970404 | 0.16263667 | -0.37787596 |
| 1096 | -0.46753108 | 0.23277295 | -0.335641444 |
| 1097 | -1.68253828 | -0.77835842 | -1.053628216 |
| 1098 | -1.24681156 | -0.453978125 | -0.797874758 |
| 1099 | -0.8404126 | -0.068228585 | -0.556199472 |
| 1100 | -1.18815604 | -0.372152465 | -0.762679328 |
| 1101 | -1.69091764 | -0.76082435 | -1.074745474 |
| 1102 | -2.6838718 | -1.657984265 | -1.675414146 |
| 1103 | -1.66996924 | -0.76082435 | -1.055974578 |
| 1104 | -0.48010012 | 0.2035495 | -0.34268053 |
| 1105 | -0.43401364 | 0.23277295 | -0.309831462 |
| 1106 | -0.43401364 | 0.276608125 | -0.309831462 |
| 1107 | -2.03866108 | -1.102738715 | -1.283571692 |
| 1108 | -1.16301796 | -0.325394945 | -0.746254794 |
| 1109 | -1.74538348 | -0.839727665 | -1.100555456 |
| 1110 | -1.72862476 | -0.83680532 | -1.095862732 |
| 1111 | -1.0498966 | -0.28155977 | -0.680556658 |
| 1112 | -0.1491154 | 0.510395725 | -0.147932484 |
| 1113 | -0.14492572 | 0.527929795 | -0.138547036 |
| 1114 | -0.92001652 | -0.179277695 | -0.617204884 |
| 1115 | -0.39211684 | 0.247384675 | -0.291060566 |
| 1116 | -0.337651 | 0.37304551 | -0.255865136 |
| 1117 | -0.80689516 | -0.050694515 | -0.551506748 |
| 1118 | -2.12664436 | -1.085204645 | -1.349269828 |
| 1119 | -1.47724396 | -0.529959095 | -0.948041926 |
| 1120 | -0.43401364 | 0.2619964 | -0.321563272 |
| 1121 | -0.79432612 | -0.06530624 | -0.544467662 |
| 1122 | -0.47172076 | 0.25615171 | -0.35441234 |
| 1123 | -0.39211684 | 0.38473489 | -0.29575329 |
| 1124 | -0.45077236 | 0.25615171 | -0.326255996 |
| 1125 | -1.04570692 | -0.243569285 | -0.689942106 |
| 1126 | -1.0917934 | -0.24064694 | -0.727483898 |
| 1127 | -0.12397732 | 0.5542309 | -0.145586122 |
| 1128 | -0.24966772 | 0.481172275 | -0.218323344 |
| 1129 | -2.03028172 | -1.073515265 | -1.283571692 |
| 1130 | -1.18815604 | -0.290326805 | -0.78848931 |
| 1131 | -1.12531084 | -0.237724595 | -0.739215708 |
| 1132 | -2.42411164 | -1.377439145 | -1.522900616 |
| 1133 | -2.27747284 | -1.243011275 | -1.431392498 |
| 1134 | -0.88649908 | -0.0302381 | -0.596087626 |
| 1135 | -2.21462764 | -1.19333141 | -1.407928878 |
| 1136 | -1.2593806 | -0.395531225 | -0.833070188 |
| 1137 | -0.00666628 | 0.603910765 | -0.065809814 |
| 1138 | -1.489813 | -0.681921035 | -0.94100284 |
| 1139 | -1.12950052 | -0.407220605 | -0.74156207 |
| 1140 | -2.39897356 | -1.465109495 | -1.508822444 |
| 1141 | -0.70634284 | -0.091607345 | -0.478769526 |
| 1142 | -0.79013644 | -0.09452969 | -0.525696766 |
| 1143 | 0.24052484 | 0.73541629 | 0.098435526 |
| 1144 | -0.26642644 | 0.335055025 | -0.211284258 |
| 1145 | 0.605027 | 0.995504995 | 0.304915382 |
| 1146 | 0.67206188 | 1.048107205 | 0.351842622 |
| 1147 | 0.1022654 | 0.57176497 | 0.013966494 |
| 1148 | 0.0603686 | 0.54254152 | -0.004804402 |
| 1149 | -0.28318516 | 0.27953047 | -0.220669706 |
| 1150 | 0.09807572 | 0.50747338 | 0.004581046 |
| 1151 | -2.10988564 | -1.190409065 | -1.321113484 |
| 1152 | -1.4479162 | -0.676076345 | -0.922231944 |
| 1153 | -0.09045988 | 0.416880685 | -0.103351606 |
| 1154 | -0.63930796 | -0.027315755 | -0.427149562 |
| 1155 | 0.3117494 | 0.738338635 | 0.154748214 |
| 1156 | 0.33688748 | 0.75295036 | 0.166480024 |
| 1157 | 1.11197828 | 1.352031085 | 0.624020614 |
| 1158 | 0.01428212 | 0.498706345 | -0.032960746 |
| 1159 | -1.175587 | -0.47443454 | -0.755640242 |
| 1160 | 0.91087364 | 1.13285521 | 0.492624342 |
| 1161 | 0.24471452 | 0.633134215 | 0.096089164 |
| 1162 | 0.56731988 | 0.884455885 | 0.297876296 |
| 1163 | 1.47229076 | 1.574129305 | 0.828154108 |
| 1164 | 0.44162948 | 0.79970788 | 0.218099988 |
| 1165 | -1.68672796 | -0.921553325 | -1.065360026 |
| 1166 | -0.88649908 | -0.243569285 | -0.582009454 |
| 1167 | -1.6155034 | -0.851417045 | -1.044242768 |
| 1168 | -0.69796348 | -0.10037438 | -0.476423164 |
| 1169 | -1.94229844 | -1.09981637 | -1.220219918 |
| 1170 | -0.99543076 | -0.377997155 | -0.6594394 |
| 1171 | -3.12797788 | -2.067112565 | -1.947592138 |
| 1172 | -1.66996924 | -0.85433939 | -1.060667302 |
| 1173 | -0.86555068 | -0.24064694 | -0.57731673 |
| 1174 | -0.16168444 | 0.28537516 | -0.159664294 |
| 1175 | -0.61416988 | -0.114986105 | -0.443574096 |
| 1176 | -2.8933558 | -1.839169655 | -1.799771332 |
| 1177 | -2.03028172 | -1.18748672 | -1.274186244 |
| 1178 | -2.06379916 | -1.24593362 | -1.297649864 |
| 1179 | -0.64768732 | -0.15297659 | -0.457652268 |
| 1180 | -0.80689516 | -0.255258665 | -0.55385311 |
| 1181 | -0.966103 | -0.430599365 | -0.63597578 |
| 1182 | -1.43534716 | -0.74913497 | -0.91753922 |
| 1183 | 0.30337004 | 0.56592028 | 0.117206422 |
| 1184 | 0.13578284 | 0.43149241 | 0.011620132 |
| 1185 | -1.44372652 | -0.76666904 | -0.931617392 |
| 1186 | 0.44581916 | 0.59514373 | 0.19698273 |
| 1187 | 0.290801 | 0.46656055 | 0.100781888 |
| 1188 | -0.5261866 | -0.09452969 | -0.382568684 |
| 1189 | -2.72995828 | -1.80117917 | -1.705916852 |
| 1190 | -1.61131372 | -0.95369912 | -1.030164596 |
| 1191 | 0.45419852 | 0.633134215 | 0.213407264 |
| 1192 | -0.13235668 | 0.171403705 | -0.136200674 |
| 1193 | -0.80270548 | -0.30493853 | -0.546814024 |
| 1194 | 0.13159316 | 0.34382206 | 0.016312856 |
| 1195 | 0.0184718 | 0.25030702 | -0.047038918 |
| 1196 | -0.14492572 | 0.112956805 | -0.140893398 |
| 1197 | 0.04360988 | 0.27953047 | -0.028268022 |
| 1198 | 2.10074276 | 1.807916905 | 1.22938201 |
| 1199 | 1.63987796 | 1.451390815 | 0.950164932 |
| 1200 | 0.11902412 | 0.31459861 | 0.016312856 |
| 1201 | 1.07008148 | 1.036417825 | 0.588825184 |
| 1202 | -0.22033996 | 0.02236411 | -0.176088828 |
| 1203 | 0.7516658 | 0.74710567 | 0.417540758 |
| 1204 | 0.86059748 | 0.82893133 | 0.49027798 |
| 1205 | -0.22871932 | -0.01270403 | -0.171396104 |
| 1206 | -1.14206956 | -0.664386965 | -0.727483898 |
| 1207 | -0.89487844 | -0.512425025 | -0.582009454 |
| 1208 | -0.15330508 | 0.031131145 | -0.138547036 |
| 1209 | -1.42277812 | -0.874795805 | -0.903461048 |
| 1210 | -0.06113212 | 0.10418977 | -0.0728489 |
| 1211 | -0.0443734 | 0.12756853 | -0.06111709 |
| 1212 | -0.09045988 | 0.09250039 | -0.091619796 |
| 1213 | -1.908781 | -1.196253755 | -1.189717212 |
| 1214 | -1.48143364 | -0.94785443 | -0.94100284 |
| 1215 | -0.04018372 | 0.130490875 | -0.058770728 |
| 1216 | 0.19024868 | 0.30875392 | 0.084357354 |
| 1217 | -0.81108484 | -0.448133435 | -0.521004042 |
| 1218 | -0.52199692 | -0.220190525 | -0.35441234 |
| 1219 | 1.2125306 | 1.018883755 | 0.696757836 |
| 1220 | -0.11559796 | 0.048665215 | -0.115083416 |
| 1221 | -0.18263284 | -0.04192748 | -0.152625208 |
| 1222 | -0.13654636 | -0.03608279 | -0.115083416 |
| 1223 | -1.19234572 | -0.79589249 | -0.755640242 |
| 1224 | -0.13654636 | -0.021471065 | -0.117429778 |
| 1225 | 0.72233804 | 0.592221385 | 0.41988712 |
| 1226 | -0.29994388 | -0.150054245 | -0.204245172 |
| 1227 | -0.01085596 | 0.06327694 | -0.021228936 |
| 1228 | 0.21538676 | 0.200627155 | 0.105474612 |
| 1229 | -0.76918804 | -0.48027923 | -0.49519406 |
| 1230 | -0.39211684 | -0.19973411 | -0.26055786 |
| 1231 | -2.47019812 | -1.710586475 | -1.544017874 |
| 1232 | -1.68672796 | -1.16995265 | -1.055974578 |
| 1233 | -0.99962044 | -0.687765725 | -0.640668504 |
| 1234 | -0.9451546 | -0.664386965 | -0.61251216 |
| 1235 | -0.6309286 | -0.424754675 | -0.410725028 |
| 1236 | -0.8194642 | -0.54457082 | -0.513964956 |
| 1237 | -1.61969308 | -1.12319513 | -1.013740062 |
| 1238 | -0.75242932 | -0.51534737 | -0.49519406 |
| 1239 | -0.38792716 | -0.290326805 | -0.26055786 |
| 1240 | -0.547135 | -0.40429826 | -0.359105064 |
| 1241 | -0.442393 | -0.30493853 | -0.298099652 |
| 1242 | -2.22719668 | -1.57323626 | -1.38681162 |
| 1243 | -0.73986028 | -0.5562602 | -0.485808612 |
| 1244 | 0.19443836 | 0.089578045 | 0.10312825 |
| 1245 | 1.0868402 | 0.68281408 | 0.661562406 |
| 1246 | -0.7356706 | -0.56794958 | -0.488154974 |
| 1247 | 0.49190564 | 0.294142195 | 0.2791054 |
| 1248 | -1.2174838 | -0.939087395 | -0.779103862 |
| 1249 | 0.76004516 | 0.340899715 | 0.412848034 |
| 1250 | -2.4953362 | -1.786567445 | -1.579213304 |
| 1251 | -1.5107614 | -1.155340925 | -0.97619827 |
| 1252 | -0.44658268 | -0.40429826 | -0.300446014 |
| 1253 | -0.56389372 | -0.500735645 | -0.368490512 |
| 1254 | 1.03237436 | 0.516240415 | 0.624020614 |
| 1255 | 0.56731988 | 0.20939419 | 0.328379002 |
| 1256 | -0.2957542 | -0.36338543 | -0.192513362 |
| 1257 | 0.85221812 | 0.305831575 | 0.52547341 |
| 1258 | 0.4583882 | 0.095422735 | 0.276759038 |
| 1259 | 0.65530316 | 0.188937775 | 0.403462586 |
| 1260 | -2.19367924 | -1.669673645 | -1.363348 |
| 1261 | -0.73986028 | -0.687765725 | -0.452959544 |
| 1262 | 0.71395868 | 0.13925791 | 0.450389826 |
| 1263 | 0.19862804 | -0.11206376 | 0.13832368 |
| 1264 | 0.93182204 | 0.39057958 | 0.57240065 |
| 1265 | 1.62730892 | 0.7880185 | 0.975974914 |
| 1266 | 0.5212334 | -0.00101465 | 0.328379002 |
| 1267 | 0.13997252 | -0.202656455 | 0.074971906 |
| 1268 | 0.63854444 | 0.095422735 | 0.368267156 |
| 1269 | 2.41915844 | 1.13285521 | 1.485135468 |
| 1270 | 0.24052484 | -0.2055788 | 0.140670042 |
| 1271 | 2.14682924 | 0.97504858 | 1.327929214 |
| 1272 | 0.86897684 | 0.19186012 | 0.555976116 |
| 1273 | -1.51495108 | -1.36282742 | -0.936310116 |
| 1274 | 0.29918036 | -0.25233632 | 0.213407264 |
| 1275 | 1.5267566 | 0.53669683 | 0.966589466 |
| 1276 | 1.6314986 | 0.62436718 | 1.02994124 |
| 1277 | 1.5058082 | 0.586376695 | 0.938433122 |
| 1278 | 0.13159316 | -0.255258665 | 0.077318268 |
| 1279 | -1.66158988 | -1.31022521 | -1.053628216 |
| 1280 | -0.58484212 | -0.600095375 | -0.368490512 |
| 1281 | 0.70138964 | 0.112956805 | 0.44335074 |
| 1282 | 0.89411492 | 0.457793515 | 0.555976116 |
| 1283 | -0.82784356 | -0.77835842 | -0.525696766 |
| 1284 | -0.35440972 | -0.39845357 | -0.255865136 |
| 1285 | -1.22167348 | -0.76082435 | -0.734522984 |
| 1286 | -0.35440972 | -0.37507481 | -0.244133326 |
| 1287 | -1.37250196 | -0.9069416 | -0.887036514 |
| 1288 | -0.57646276 | -0.34585136 | -0.410725028 |
| 1289 | -0.9032578 | -0.746212625 | -0.593741264 |
| 1290 | 0.66368252 | 0.399346615 | 0.365920794 |
| 1291 | 2.41915844 | 1.579973995 | 1.426476418 |
| 1292 | 0.84383876 | 0.48993931 | 0.476199808 |
| 1293 | 0.30755972 | 0.28537516 | 0.17351911 |
| 1294 | 0.35783588 | 0.0282088 | 0.229831798 |
| 1295 | 1.1706338 | 0.726649255 | 0.696757836 |
| 1296 | -0.07789084 | -0.45690047 | -0.070502538 |
| 1297 | 0.13159316 | -0.208501145 | 0.023351942 |
| 1298 | -1.54427884 | -1.27515707 | -0.959773736 |
| 1299 | -0.547135 | -0.53288144 | -0.38960777 |
| 1300 | 0.30755972 | -0.044849825 | 0.152401852 |
| 1301 | -0.43820332 | -0.769591385 | -0.267596946 |
| 1302 | 0.32012876 | -0.208501145 | 0.171172748 |
| 1303 | -1.06665532 | -1.260545345 | -0.6594394 |
| 1304 | -0.34184068 | -0.71406683 | -0.199552448 |
| 1305 | -0.22452964 | -0.56210489 | -0.140893398 |
| 1306 | 0.08550668 | -0.28740446 | 0.049161924 |
| 1307 | -0.01085596 | -0.266948045 | -0.000111678 |
| 1308 | 0.26147324 | -0.26987039 | 0.171172748 |
| 1309 | 1.49323916 | 0.57176497 | 0.954857656 |
| 1310 | 0.09807572 | -0.693610415 | 0.119552784 |
| 1311 | -0.76080868 | -0.962466155 | -0.474076802 |
| 1312 | -2.75090668 | -2.12848181 | -1.717648662 |
| 1313 | -0.04018372 | -0.646852895 | -0.007150764 |
| 1314 | 1.52256692 | 0.475327585 | 0.943125846 |
| 1315 | 2.46524492 | 1.112398795 | 1.52267726 |
| 1316 | 2.6579702 | 1.28481715 | 1.647034446 |
| 1317 | 0.081317 | -0.39260888 | 0.049161924 |
| 1318 | -0.36278908 | -0.553337855 | -0.23709424 |
| 1319 | -2.22719668 | -1.833324965 | -1.393850706 |
| 1320 | -0.76080868 | -0.93616505 | -0.474076802 |
| 1321 | 0.34526684 | -0.15882128 | 0.192290006 |
| 1322 | 0.72652772 | 0.294142195 | 0.450389826 |
| 1323 | 0.31593908 | 0.0282088 | 0.171172748 |
| 1324 | 0.081317 | -0.4393664 | 0.016312856 |
| 1325 | 0.11483444 | -0.477356885 | 0.0444692 |
| 1326 | -0.89487844 | -0.646852895 | -0.572624006 |
| 1327 | -0.7147222 | -0.792970145 | -0.462344992 |
| 1328 | 0.85221812 | 0.580532005 | 0.520780686 |
| 1329 | -0.128167 | -0.18220004 | -0.091619796 |
| 1330 | -2.1601618 | -1.377439145 | -1.349269828 |
| 1331 | -0.74404996 | -0.500735645 | -0.48346225 |
| 1332 | -0.17844316 | -0.208501145 | -0.117429778 |
| 1333 | -2.13502372 | -1.418351975 | -1.368040724 |
| 1334 | -2.54561236 | -1.70181944 | -1.626140544 |
| 1335 | -1.08760372 | -0.483201575 | -0.631283056 |
| 1336 | 0.52961276 | 0.51331807 | 0.316647192 |
| 1337 | -0.25804708 | -0.068228585 | -0.194859724 |
| 1338 | -0.0862702 | 0.072043975 | -0.115083416 |
| 1339 | 1.9247762 | 1.445546125 | 1.114410272 |
| 1340 | 2.3018474 | 1.731935935 | 1.327929214 |
| 1341 | 1.93734524 | 1.357875775 | 1.163683874 |
| 1342 | 3.62578628 | 2.579415985 | 2.240664032 |
| 1343 | 2.01275948 | 1.2555937 | 1.245806544 |
| 1344 | 0.72652772 | 0.522085105 | 0.422233482 |
| 1345 | 2.84231612 | 1.88682022 | 1.762006184 |
| 1346 | 1.64406764 | 1.00427203 | 1.020555792 |
| 1347 | 1.42620428 | 0.85815478 | 0.884466796 |
| 1348 | 1.57703276 | 0.89906761 | 0.975974914 |
| 1349 | -1.02475852 | -0.7316009 | -0.67117121 |
| 1350 | 1.69015412 | 0.99258265 | 1.04167305 |
| 1351 | 0.4793366 | 0.24446233 | 0.300222658 |
| 1352 | -0.8823094 | -0.83096063 | -0.567931282 |
| 1353 | -1.41439876 | -1.102738715 | -0.901114686 |
| 1354 | -0.20358124 | -0.35169605 | -0.138547036 |
| 1355 | 0.54637148 | 0.048665215 | 0.33776445 |
| 1356 | 1.87450004 | 0.7880185 | 1.154298426 |
| 1357 | 1.21672028 | 0.41395834 | 0.743685076 |
| 1358 | 0.32012876 | 0.12172384 | 0.192290006 |
| 1359 | 2.2180538 | 1.080253 | 1.374856454 |
| 1360 | 1.3172726 | 0.697425805 | 0.792958678 |
| 1361 | -0.11978764 | -0.331239635 | -0.09631252 |
| 1362 | 2.3018474 | 1.09194238 | 1.414744608 |
| 1363 | 2.15939828 | 1.030573135 | 1.349046472 |
| 1364 | 0.4793366 | 0.05743225 | 0.288490848 |
| 1365 | 0.91087364 | 0.51331807 | 0.541897944 |
| 1366 | 0.59245796 | 0.19186012 | 0.342457174 |
| 1367 | 1.50161852 | 1.080253 | 0.903237692 |
| 1368 | 0.4793366 | 0.358433785 | 0.311954468 |
| 1369 | 2.63283212 | 1.22052556 | 1.623570826 |
| 1370 | 3.55037204 | 1.82837332 | 2.184351344 |
| 1371 | 1.2963242 | 0.51331807 | 0.776534144 |
| 1372 | 0.00590276 | -0.28740446 | -0.00245804 |
| 1373 | 0.21119708 | -0.144209555 | 0.09139644 |
| 1374 | 0.46257788 | 0.13925791 | 0.222792712 |
| 1375 | 1.128737 | 0.3204433 | 0.685026026 |
| 1376 | -1.594555 | -1.260545345 | -1.0113937 |
| 1377 | -1.23424252 | -1.1407292 | -0.807260206 |
| 1378 | -0.23709868 | -0.27571508 | -0.162010656 |
| 1379 | 0.17348996 | -0.348773705 | 0.107820974 |
| 1380 | 0.9820982 | 0.235695295 | 0.591171546 |
| 1381 | 0.1441622 | -0.34585136 | 0.06793282 |
| 1382 | -0.30832324 | -0.6147071 | -0.218323344 |
| 1383 | 0.07712732 | -0.050694515 | 0.006927408 |
| 1384 | 0.00590276 | -0.383841845 | -0.047038918 |
| 1385 | -0.56389372 | -0.722833865 | -0.406032304 |
| 1386 | 0.41230172 | 0.0282088 | 0.180558196 |
| 1387 | 1.04075372 | 0.34966675 | 0.626366976 |
| 1388 | 2.34793388 | 1.11532114 | 1.42882278 |
| 1389 | 1.6105502 | 0.650668285 | 0.973628552 |
| 1390 | 0.07712732 | -0.208501145 | 0.018659218 |
| 1391 | -0.69796348 | -0.70237745 | -0.525696766 |
| 1392 | 0.22376612 | 0.06912163 | 0.089050078 |
| 1393 | -0.15330508 | -0.424754675 | -0.152625208 |
| 1394 | -1.07922436 | -0.997534295 | -0.70636664 |
| 1395 | 0.30337004 | -0.17051066 | 0.143016404 |
| 1396 | -0.22871932 | -0.44521109 | -0.185474276 |
| 1397 | -1.79984932 | -1.45049777 | -1.206141746 |
| 1398 | -1.11693148 | -0.98876726 | -0.727483898 |
| 1399 | 1.11616796 | 0.25615171 | 0.635752424 |
| 1400 | 1.10359892 | 0.39642427 | 0.593517908 |
| 1401 | -0.23709868 | -0.57379427 | -0.180781552 |
| 1402 | 1.30051388 | 0.23277295 | 0.776534144 |
| 1403 | 1.757189 | 0.71788222 | 1.069829394 |
| 1404 | 0.78937292 | 0.18017074 | 0.436311654 |
| 1405 | 1.19996156 | 0.416880685 | 0.72491418 |
| 1406 | -1.37250196 | -1.21086548 | -0.891729238 |
| 1407 | -0.87811972 | -0.915708635 | -0.56558492 |
| 1408 | -1.23005284 | -1.10566106 | -0.767372052 |
| 1409 | -0.95353396 | -0.93032036 | -0.596087626 |
| 1410 | -0.34184068 | -0.524114405 | -0.22536243 |
| 1411 | 0.57150956 | 0.25030702 | 0.34949626 |
| 1412 | 0.2698526 | -0.044849825 | 0.171172748 |
| 1413 | 1.0449434 | 0.288297505 | 0.65452332 |
| 1414 | -1.35993292 | -0.93032036 | -0.85888017 |
| 1415 | -1.0289482 | -0.687765725 | -0.650053952 |
| 1416 | -2.41992196 | -1.81286855 | -1.525246978 |
| 1417 | -2.81794156 | -1.833324965 | -1.766922264 |
| 1418 | -1.53589948 | -1.453420115 | -0.943349202 |
| 1419 | -1.51495108 | -1.394973215 | -0.924578306 |
| 1420 | -1.61131372 | -1.50309998 | -1.013740062 |
| 1421 | -0.07370116 | -0.652697585 | -0.030614384 |
| 1422 | -0.08208052 | -0.5562602 | -0.063463452 |
| 1423 | -1.24262188 | -0.95954381 | -0.743908432 |
| 1424 | 0.19024868 | -0.37507481 | 0.121899146 |
| 1425 | -1.79984932 | -1.447575425 | -1.131058162 |
| 1426 | -1.47724396 | -1.131962165 | -0.931617392 |
| 1427 | 0.75585548 | 0.153869635 | 0.518434324 |
| 1428 | 0.94439108 | 0.235695295 | 0.67798694 |
| 1429 | 0.48352628 | 0.01067473 | 0.286144486 |
| 1430 | -1.3641226 | -1.08812699 | -0.865919256 |
| 1431 | -0.8194642 | -0.851417045 | -0.509272232 |
| 1432 | -0.1700638 | -0.45690047 | -0.08458071 |
| 1433 | 0.56731988 | -0.085762655 | 0.351842622 |
| 1434 | 0.34107716 | -0.138364865 | 0.199329092 |
| 1435 | 0.21957644 | -0.307860875 | 0.105474612 |
| 1436 | 1.7152922 | 0.62436718 | 1.04167305 |
| 1437 | 0.5212334 | -0.085762655 | 0.300222658 |
| 1438 | 1.41363524 | 0.446104135 | 0.856310452 |
| 1439 | 0.02266148 | -0.34585136 | 0.046815562 |
| 1440 | -0.71053252 | -0.83096063 | -0.474076802 |
| 1441 | -0.4214446 | -0.792970145 | -0.3074851 |
| 1442 | -1.22586316 | -1.32775928 | -0.81195293 |
| 1443 | -2.7886138 | -2.037889115 | -1.736419558 |
| 1444 | -1.05827596 | -1.27515707 | -0.699327554 |
| 1445 | -0.61835956 | -0.42767702 | -0.384915046 |
| 1446 | 1.56446372 | 0.750028015 | 0.919662226 |
| 1447 | 2.37307196 | 1.129932865 | 1.482789106 |
| 1448 | 1.89125876 | 0.200627155 | 1.151952064 |
| 1449 | -0.47591044 | 0.20939419 | -0.326255996 |
| 1450 | 1.42620428 | 0.01067473 | 0.884466796 |
| 1451 | 2.50295204 | 0.78217381 | 1.55787269 |
| 1452 | 1.30470356 | 0.7880185 | 0.72491418 |
| 1453 | -1.25519092 | -0.52703675 | -0.950388288 |
| 1454 | -0.66025636 | -0.091607345 | -0.55385311 |
| 1455 | -0.70634284 | 0.02236411 | -0.70636664 |
| 1456 | -0.71891188 | 0.282452815 | -0.55385311 |
| 1457 | -0.09464956 | 0.59514373 | -0.124468864 |
| 1458 | 0.65949284 | 0.10418977 | 0.426926206 |
| 1459 | 0.81032132 | 0.013597075 | 0.45508255 |
| 1460 | 0.61340636 | -0.266948045 | 0.368267156 |
| 1461 | 1.15806476 | 0.282452815 | 0.71318237 |
| 1462 | -0.23709868 | 0.212316535 | -0.232401516 |
| 1463 | -0.13235668 | 0.399346615 | -0.27228967 |
| 1464 | 0.3117494 | -0.114986105 | 0.143016404 |
| 1465 | -1.07922436 | -0.190967075 | -0.72983026 |
| 1466 | -0.49685884 | -0.249413975 | -0.40133958 |
| 1467 | -0.45077236 | -0.06530624 | -0.387261408 |
| 1468 | 0.89411492 | 0.907834645 | 0.492624342 |
| 1469 | 1.1496854 | 0.855232435 | 0.464467998 |
| 1470 | 0.42068108 | 0.05158756 | 0.10312825 |
| 1471 | -1.03313788 | -0.6147071 | -0.7767575 |
| 1472 | -2.10150628 | -1.46803184 | -1.478319738 |
| 1473 | -2.77185508 | -1.59077033 | -1.77396135 |
| 1474 | 0.28661132 | -0.255258665 | 0.178211834 |
| 1475 | -0.05694244 | -0.056539205 | -0.29575329 |
| 1476 | -1.27194964 | -0.42767702 | -0.809606568 |
| 1477 | -0.19520188 | -0.01270403 | -0.28402148 |
| 1478 | -0.49266916 | -0.003936995 | -0.347373254 |
| 1479 | -0.31251292 | 0.276608125 | -0.314524186 |
| 1480 | 0.5421818 | 0.58345435 | 0.309608106 |
| 1481 | 0.11902412 | 0.335055025 | 0.192290006 |
| 1482 | -0.66025636 | -0.699455105 | -0.434188648 |
| 1483 | -1.18396636 | -0.974155535 | -0.75329388 |
| 1484 | -1.8668842 | -1.237166585 | -1.201449022 |
| 1485 | -2.73414796 | -1.66090661 | -1.719995024 |
| 1486 | -0.76918804 | -0.266948045 | -0.5421213 |
| 1487 | 0.12740348 | 0.41980303 | 0.135977318 |
| 1488 | -0.36697876 | 0.253229365 | -0.26055786 |
| 1489 | -0.1700638 | 0.142180255 | -0.086927072 |
| 1490 | -0.128167 | 0.43149241 | -0.02592166 |
| 1491 | -1.72443508 | -1.26931238 | -1.15217542 |
| 1492 | -1.28870836 | -0.73744559 | -0.781450224 |
| 1493 | -1.87945324 | -1.447575425 | -1.231951728 |
| 1494 | -1.46048524 | -0.968310845 | -0.924578306 |
| 1495 | -1.3431742 | -0.69653276 | -0.877651066 |
| 1496 | -0.59741116 | -0.144209555 | -0.281675118 |
| 1497 | -0.79013644 | -0.26987039 | -0.452959544 |
| 1498 | -0.6937738 | -0.442288745 | -0.391954132 |
| 1499 | -1.51495108 | -0.863106425 | -0.879997428 |
| 1500 | -1.09598308 | -0.559182545 | -0.598433988 |
| 1501 | -0.76499836 | -0.14128721 | -0.523350404 |
| 1502 | -0.0443734 | 0.282452815 | -0.004804402 |
| 1503 | -2.21462764 | -1.301458175 | -1.382118896 |
| 1504 | -1.97581588 | -1.324836935 | -1.229605366 |
| 1505 | -0.25804708 | -0.413065295 | -0.321563272 |
| 1506 | 0.12740348 | -0.074073275 | -0.000111678 |
| 1507 | -0.36278908 | -0.337084325 | -0.173742466 |
| 1508 | 0.186059 | 0.165559015 | 0.124245508 |
| 1509 | -0.21615028 | -0.249413975 | -0.157317932 |
| 1510 | -0.21615028 | -0.348773705 | -0.082234348 |
| 1511 | -0.20777092 | 0.112956805 | -0.145586122 |
| 1512 | -0.18682252 | 0.206471845 | -0.152625208 |
| 1513 | -2.42830132 | -1.470954185 | -1.51586153 |
| 1514 | -2.22719668 | -1.15826327 | -1.403236154 |
| 1515 | -1.2174838 | -0.5562602 | -0.76502569 |
| 1516 | 0.39973268 | 0.40226896 | 0.316647192 |
| 1517 | -2.05123012 | -0.863106425 | -1.271839882 |
| 1518 | -0.59322148 | 0.09250039 | -0.368490512 |
| 1519 | 1.2125306 | 0.311676265 | 0.722567818 |
| 1520 | 1.56027404 | 0.311676265 | 0.804690488 |
| 1521 | 1.8200342 | 0.539619175 | 1.044019412 |
| 1522 | 1.41363524 | 0.44902648 | 0.77184142 |
| 1523 | -0.71891188 | -0.78420311 | -0.549160386 |
| 1524 | 0.86059748 | 0.107112115 | 0.401116224 |
| 1525 | 0.07712732 | -0.31662791 | -0.035307108 |
| 1526 | 2.06303564 | 0.814319605 | 1.144912978 |
| 1527 | 1.70691284 | 0.598066075 | 1.00647762 |
| 1528 | 1.97505236 | 0.58929904 | 1.166030236 |
| 1529 | 1.84517228 | 0.627289525 | 1.08860029 |
| 1530 | 1.77394772 | 0.586376695 | 1.058097584 |
| 1531 | -0.89906812 | -0.63808586 | -0.61251216 |
| 1532 | -2.118265 | -1.570313915 | -1.370387086 |
| 1533 | -0.10302892 | -0.53872613 | -0.098658882 |
| 1534 | -0.0653218 | -0.366307775 | -0.042346194 |
| 1535 | -1.69510732 | -1.49725529 | -1.133404524 |
| 1536 | -0.26642644 | -0.7316009 | -0.197206086 |
| 1537 | 1.50161852 | 0.235695295 | 0.88915952 |
| 1538 | -0.50104852 | -0.83680532 | -0.340334168 |
| 1539 | 0.29918036 | -0.430599365 | 0.11486006 |
| 1540 | 0.186059 | -0.46274516 | 0.077318268 |
| 1541 | -1.66996924 | -1.47387653 | -1.063013664 |
| 1542 | 0.74328644 | -0.126675485 | 0.424579844 |
| 1543 | 0.7935626 | -0.17635535 | 0.483238894 |
| 1544 | 1.6943438 | 0.495784 | 1.015863068 |
| 1545 | 1.98343172 | 0.714959875 | 1.184801132 |
| 1546 | -0.6728254 | -0.87771815 | -0.44826682 |
| 1547 | -0.56389372 | -0.94200974 | -0.382568684 |
| 1548 | -0.63511828 | -0.79589249 | -0.427149562 |
| 1549 | -0.26223676 | -0.6147071 | -0.192513362 |
| 1550 | 0.55056116 | -0.132520175 | 0.311954468 |
| 1551 | 0.59664764 | -0.220190525 | 0.33776445 |
| 1552 | 0.55475084 | -0.097452035 | 0.311954468 |
| 1553 | 1.35079004 | 0.411035995 | 0.816422298 |
| 1554 | -0.67701508 | -0.284482115 | -0.469384078 |
| 1555 | -0.47172076 | -0.693610415 | -0.298099652 |
| 1556 | -0.24128836 | -0.623474135 | -0.157317932 |
| 1557 | -0.84460228 | -0.87771815 | -0.53038949 |
| 1558 | -0.45496204 | -0.681921035 | -0.288714204 |
| 1559 | -0.15749476 | -0.53288144 | -0.098658882 |
| 1560 | 0.44162948 | -0.22895756 | 0.276759038 |
| 1561 | 1.10359892 | 0.183093085 | -0.476423164 |
| 1562 | 1.09940924 | 0.2619964 | -0.452959544 |
| 1563 | 0.47514692 | -0.144209555 | -0.666478486 |
| 1564 | -0.37535812 | -0.66146462 | -0.91753922 |
| 1565 | -0.01504564 | -0.39845357 | -0.826031102 |
| 1566 | -0.37535812 | -0.617629445 | -0.945695564 |
| 1567 | -0.01085596 | -0.436444055 | -0.81195293 |
| 1568 | -0.07370116 | -0.4978133 | -0.722791174 |
| 1569 | -0.58484212 | -0.75497966 | -0.879997428 |
| 1570 | 1.07846084 | 0.218161225 | -0.352065978 |
| 1571 | 0.96952916 | 0.142180255 | -0.462344992 |
| 1572 | 0.3745946 | -0.24649163 | -0.68290302 |
| 1573 | 0.65530316 | -0.09452969 | -0.579663092 |
| 1574 | 0.54637148 | -0.074073275 | -0.490501336 |
| 1575 | 0.47514692 | -0.097452035 | -0.640668504 |
| 1576 | 0.53799212 | -0.109141415 | -0.701673916 |
| 1577 | 0.7935626 | -0.003936995 | -0.725137536 |
| 1578 | 0.97790852 | 0.17432605 | -0.591394902 |
| 1579 | 0.9611498 | 0.19186012 | -0.434188648 |
| 1580 | 1.52256692 | 0.510395725 | -0.164357018 |
| 1581 | 1.75299932 | 0.61852249 | -0.044692556 |
| 1582 | 1.27118612 | 0.34382206 | -0.333295082 |
| 1583 | 0.69301028 | -0.003936995 | -0.560892196 |
| 1584 | 0.51285404 | -0.138364865 | -0.643014866 |
| 1585 | 1.93315556 | 0.691581115 | -0.126815226 |
| 1586 | 2.16358796 | 0.84062071 | -0.147932484 |
| 1587 | 1.73205092 | 0.65943532 | -0.206591534 |
| 1588 | 2.02113884 | 0.82893133 | -0.023575298 |
| 1589 | 2.53646948 | 1.112398795 | 0.06793282 |
| 1590 | -0.11559796 | -0.48612392 | -0.872958342 |
| 1591 | -1.0917934 | -1.091049335 | -1.21083447 |
| 1592 | 0.11902412 | -0.37507481 | -0.809606568 |
| 1593 | 0.65111348 | -0.044849825 | -0.652400314 |
| 1594 | 0.6888206 | -0.015626375 | -0.56558492 |
| 1595 | 0.77680388 | 0.05743225 | -0.539774938 |
| 1596 | 1.74043028 | 0.54254152 | -0.305138738 |
| 1597 | 2.54903852 | 1.06271893 | 0.028044666 |
| 1598 | 2.48619332 | 1.042262515 | -0.044692556 |
| 1599 | 0.6469238 | -0.08284031 | -0.725137536 |
| 1600 | 0.290801 | -0.30493853 | -0.802567482 |
| 1601 | -0.20358124 | -0.56794958 | -0.971505546 |
| 1602 | 0.13997252 | -0.424754675 | -0.877651066 |
| 1603 | -0.12397732 | -0.600095375 | -0.978544632 |
| 1604 | 0.08550668 | -0.42183233 | -0.720444812 |
| 1605 | 0.40392236 | -0.29324915 | -0.652400314 |
| 1606 | -0.9870514 | -1.07059292 | -1.124019076 |
| 1607 | 0.70557932 | -0.16466597 | -0.455305906 |
| 1608 | 0.99466724 | 0.0282088 | -0.361451426 |
| 1609 | 0.6469238 | -0.13544252 | -0.403685942 |
| 1610 | -1.6364518 | -1.535245775 | -1.248376262 |
| 1611 | -1.04570692 | -1.09981637 | -1.100555456 |
| 1612 | 0.15673124 | -0.354618395 | -0.631283056 |
| 1613 | -0.6728254 | -0.868951115 | -0.809606568 |
| 1614 | -0.04856308 | -0.4978133 | -0.591394902 |
| 1615 | -0.63511828 | -0.851417045 | -1.135750886 |
| 1616 | -2.60845756 | -2.01158801 | -1.823234952 |
| 1617 | -1.43534716 | -1.295613485 | -1.302342588 |
| 1618 | -0.46753108 | -0.746212625 | -0.868265618 |
| 1619 | -1.10855212 | -1.24593362 | -1.067706388 |
| 1620 | 0.32850812 | -0.36923012 | -0.422456838 |
| 1621 | 0.15254156 | -0.494890955 | -0.521004042 |
| 1622 | 0.19443836 | -0.48027923 | -0.455305906 |
| 1623 | 0.72652772 | -0.202656455 | -0.267596946 |
| 1624 | 0.06455828 | -0.55041551 | -0.699327554 |
| 1625 | -1.94229844 | -1.254700655 | -1.238990814 |
| 1626 | -1.2384322 | -0.874795805 | -0.7767575 |
| 1627 | 0.1441622 | 0.060354595 | -0.150278846 |
| 1628 | 0.03523052 | 0.16848136 | -0.286367842 |
| 1629 | -1.10855212 | -0.518269715 | -0.807260206 |
| 1630 | -2.87659708 | -1.973597525 | -1.834966762 |
| 1631 | -1.61131372 | -1.143651545 | -0.85888017 |
| 1632 | -0.35440972 | -0.337084325 | -0.370836874 |
| 1633 | 0.88573556 | 0.662357665 | 0.133630956 |
| 1634 | 0.09807572 | 0.165559015 | -0.361451426 |
| 1635 | -0.32927164 | -0.103296725 | -0.485808612 |
| 1636 | -1.42277812 | -0.81342656 | -1.208488108 |
| 1637 | -2.29004188 | -1.657984265 | -1.705916852 |
| 1638 | -1.35993292 | -0.664386965 | -1.161560868 |
| 1639 | -1.01218948 | -0.53288144 | -1.041896406 |
| 1640 | -0.16587412 | 0.33213268 | -0.450613182 |
| 1641 | -0.93677524 | -0.53872613 | -0.926924668 |
| 1642 | -1.2174838 | -0.535803785 | -1.006700976 |
| 1643 | -0.44658268 | -0.208501145 | -0.631283056 |
| 1644 | -2.59169884 | -1.652139575 | -1.724687748 |
| 1645 | -1.26357028 | -0.944932085 | -0.971505546 |
| 1646 | -0.47591044 | -0.27571508 | -0.760332966 |
| 1647 | -0.87393004 | -0.512425025 | -1.032510958 |
| 1648 | -0.74404996 | -0.38676419 | -0.966812822 |
| 1649 | 0.38297396 | 0.253229365 | -0.37787596 |
| 1650 | -1.31384644 | -0.863106425 | -1.145136334 |
| 1651 | -0.31251292 | -0.319550255 | -0.736869346 |
| 1652 | -0.91163716 | -0.68484338 | -1.142789972 |
| 1653 | -2.85564868 | -1.815790895 | -1.802117694 |
| 1654 | -1.47724396 | -0.60301772 | -1.229605366 |
| 1655 | 0.19443836 | 0.16848136 | -0.57731673 |
| 1656 | 0.12740348 | 0.12172384 | -0.62424397 |
| 1657 | -0.6937738 | -0.489046265 | -1.051281854 |
| 1658 | -1.33898452 | -0.787125455 | -1.19910266 |
| 1659 | -1.18815604 | -0.71406683 | -1.236644452 |
| 1660 | -0.46753108 | -0.39845357 | -1.016086424 |
| 1661 | -0.53456596 | -0.383841845 | -1.04658913 |
| 1662 | -1.77471124 | -1.248855965 | -1.619101458 |
| 1663 | -0.56389372 | -0.62639648 | -1.039550044 |
| 1664 | 0.11064476 | -0.114986105 | -0.983237356 |
| 1665 | -1.51914076 | -1.13488451 | -1.353962552 |
| 1666 | -0.3585994 | -0.34000667 | -0.80022112 |
| 1667 | -2.08893724 | -1.552779845 | -1.630833268 |
| 1668 | -0.58065244 | -0.442288745 | -1.09351637 |
| 1669 | 0.01009244 | 0.025286455 | -0.901114686 |
| 1670 | -2.4534394 | -1.605382055 | -1.82088859 |
| 1671 | -0.8194642 | -0.734523245 | -1.377426172 |
| 1672 | 0.00590276 | -0.11206376 | -1.03485732 |
| 1673 | 1.27118612 | 0.77632912 | -0.368490512 |
| 1674 | 1.01980532 | 0.54254152 | -0.476423164 |
| 1675 | 0.57569924 | 0.1451026 | -0.33094872 |
| 1676 | 0.85221812 | 0.422725375 | -0.490501336 |
| 1677 | -1.41858844 | -1.126117475 | -1.267147158 |
| 1678 | 0.04360988 | -0.091607345 | -0.75329388 |
| 1679 | -0.30832324 | -0.39845357 | -1.241337176 |
| 1680 | -0.05275276 | -0.202656455 | -0.767372052 |
| 1681 | 0.50866436 | 0.2035495 | -0.457652268 |
| 1682 | 0.29499068 | 0.136335565 | -0.997315528 |
| 1683 | 1.49323916 | 0.837698365 | -0.528043128 |
| 1684 | 0.97371884 | 0.50162869 | -0.551506748 |
| 1685 | -1.0079998 | -0.705299795 | -1.393850706 |
| 1686 | 1.01142596 | 0.463638205 | -0.617204884 |
| 1687 | -0.22033996 | -0.284482115 | -0.97619827 |
| 1688 | 0.50447468 | 0.01651942 | -0.614858522 |
| 1689 | 1.0658918 | 0.527929795 | -0.194859724 |
| 1690 | -0.7147222 | -0.50950268 | -0.901114686 |
| 1691 | -0.70215316 | -0.565027235 | -0.704020278 |
| 1692 | -0.07370116 | -0.18804473 | -0.772064776 |
| 1693 | 0.52542308 | 0.235695295 | -0.380222322 |
| 1694 | -0.20358124 | -0.249413975 | -0.539774938 |
| 1695 | 0.7726142 | 0.434414755 | -0.666478486 |
| 1696 | 1.5267566 | 0.93413575 | -0.438881372 |
| 1697 | 1.40944556 | 0.9049123 | -0.645361228 |
| 1698 | 0.25309388 | 0.101267425 | -0.91753922 |
| 1699 | 0.8564078 | 0.58929904 | -0.760332966 |
| 1700 | 0.70138964 | 0.47824993 | -0.722791174 |
| 1701 | 0.28242164 | 0.13341322 | -0.90580741 |
| 1702 | -0.5261866 | -0.45105578 | -1.201449022 |
| 1703 | 0.86059748 | 0.44318179 | -0.666478486 |
| 1704 | 1.10359892 | 0.70619284 | -0.567931282 |
| 1705 | 0.67206188 | 0.41395834 | -0.72983026 |
| 1706 | 0.19443836 | -0.03608279 | -0.92927103 |
| 1707 | 0.395543 | -0.179277695 | -0.84714836 |
| 1708 | 0.23214548 | 0.077888665 | -0.901114686 |
| 1709 | -0.06113212 | -0.114986105 | -1.0113937 |
| 1710 | -0.4214446 | -0.16466597 | -1.156868144 |
| 1711 | -0.50942788 | -0.18220004 | -1.217873556 |
| 1712 | -1.46048524 | -1.08812699 | -1.56278877 |
| 1713 | -0.42982396 | -0.179277695 | -1.168599954 |
| 1714 | -0.3167026 | -0.085762655 | -1.102901818 |
| 1715 | -1.99676428 | -1.42127432 | -1.788039522 |
| 1716 | -1.01218948 | -0.693610415 | -1.389157982 |
| 1717 | -0.20777092 | -0.01270403 | -1.08178456 |
| 1718 | -0.41306524 | -0.173433005 | -1.19910266 |
| 1719 | -0.01085596 | 0.06912163 | -1.009047338 |
| 1720 | -1.37250196 | -0.7900478 | -1.534632426 |
| 1721 | -0.64349764 | -0.16466597 | -1.260108072 |
| 1722 | 0.51285404 | 0.63021187 | -0.781450224 |
| 1723 | 0.30755972 | 0.40226896 | -0.875304704 |
| 1724 | -0.09464956 | 0.124646185 | -1.03485732 |
| 1725 | -0.57227308 | -0.348773705 | -1.215527194 |
| 1726 | -1.31803612 | -0.83096063 | -1.501783358 |
| 1727 | -1.17977668 | -0.70822214 | -1.473627014 |
| 1728 | -0.67701508 | -0.41598764 | -1.271839882 |
| 1729 | 0.00590276 | 0.12172384 | -1.004354614 |
| 1730 | 0.34526684 | 0.51331807 | -0.877651066 |
| 1731 | -0.84460228 | -0.44521109 | -1.32815257 |
| 1732 | -0.5261866 | 0.013597075 | -1.217873556 |
| 1733 | 0.11483444 | 0.38473489 | -0.938656478 |
| 1734 | 0.290801 | 0.259074055 | -0.889382876 |
| 1735 | 1.10359892 | 0.77632912 | -0.582009454 |
| 1736 | 0.94439108 | 0.802630225 | -0.60078035 |
| 1737 | -0.76918804 | -0.59132834 | -1.321113484 |
| 1738 | 0.39135332 | 0.19186012 | -0.875304704 |
| 1739 | 0.67206188 | 0.36720082 | -0.75329388 |
| 1740 | 0.04360988 | 0.060354595 | -1.004354614 |
| 1741 | -0.9032578 | -0.66146462 | -1.35161619 |
| 1742 | -0.59741116 | -0.50365799 | -1.22256628 |
| 1743 | 0.37878428 | 0.06912163 | -0.840109274 |
| 1744 | 0.68463092 | 0.21523888 | -0.732176622 |
| 1745 | 0.65949284 | 0.299986885 | -0.769718414 |
| 1746 | 0.02266148 | -0.237724595 | -0.99966189 |
| 1747 | 0.29499068 | -0.10037438 | -0.88234379 |
| 1748 | -0.5261866 | -0.664386965 | -1.217873556 |
| 1749 | -2.00095396 | -1.564469225 | -1.823234952 |
| 1750 | -1.01637916 | -0.91278629 | -1.414967964 |
| 1751 | -0.52199692 | -0.605940065 | -1.215527194 |
| 1752 | 0.32431844 | 0.042820525 | -0.872958342 |
| 1753 | -0.15749476 | -0.261103355 | -1.065360026 |
| 1754 | 0.86059748 | 0.37304551 | -0.652400314 |
| 1755 | 1.2753758 | 0.64190125 | -0.497540422 |
| 1756 | 0.76842452 | 0.31459861 | -0.701673916 |
| 1757 | 0.05198924 | -0.155898935 | -0.97619827 |
| 1758 | -0.55551436 | -0.471512195 | -1.217873556 |
| 1759 | -0.17844316 | -0.28155977 | -1.048935492 |
| 1760 | -1.51914076 | -1.24593362 | -1.605023286 |
| 1761 | 1.25861708 | 0.767562085 | -0.504579508 |
| 1762 | -0.7147222 | -0.68484338 | -1.264800796 |
| 1763 | 0.38297396 | 0.183093085 | -0.828377464 |
| 1764 | 0.72233804 | 0.411035995 | -0.711059364 |
| 1765 | -0.18263284 | -0.266948045 | -1.077091836 |
| 1766 | 0.92344268 | 0.54838621 | -0.626590332 |
| 1767 | 0.25728356 | 0.042820525 | -0.88234379 |
| 1768 | 0.08969636 | -0.027315755 | -0.957427374 |
| 1769 | 0.3536462 | 0.1451026 | -0.87061198 |
| 1770 | 1.28794484 | 0.71788222 | -0.50692587 |
| 1771 | 0.68044124 | 0.317520955 | -0.727483898 |
| 1772 | 0.709769 | 0.32628799 | -0.725137536 |
| 1773 | 0.37040492 | 0.11003446 | -0.844801998 |
| 1774 | 0.92344268 | 0.346744405 | -0.605473074 |
| 1775 | 0.3326978 | 0.072043975 | -0.844801998 |
| 1776 | -0.9242062 | -0.72575621 | -1.363348 |
| 1777 | -0.11140828 | -0.243569285 | -1.037203682 |
| 1778 | 0.4374398 | 0.06327694 | -0.793182034 |
| 1779 | -0.77337772 | -0.757902005 | -1.295303502 |
| 1780 | -0.36278908 | -0.471512195 | -1.10524818 |
| 1781 | -1.55684788 | -1.149496235 | -1.579213304 |
| 1782 | -0.95772364 | -0.839727665 | -1.325806208 |
| 1783 | 0.46257788 | -0.003936995 | -0.76502569 |
| 1784 | 1.32565196 | 0.533774485 | -0.450613182 |
| 1785 | 1.3172726 | 0.504551035 | -0.431842286 |
| 1786 | 0.73490708 | 0.0282088 | -0.713405726 |
| 1787 | 0.25309388 | -0.255258665 | -0.896421962 |
| 1788 | -0.18682252 | -0.44521109 | -1.063013664 |
| 1789 | -1.6155034 | -1.289768795 | -1.616755096 |
| 1790 | -0.9032578 | -0.91278629 | -1.361001638 |
| 1791 | 0.70557932 | 0.060354595 | -0.708713002 |
| 1792 | 0.35783588 | -0.15297659 | -0.856533808 |
| 1793 | 0.76004516 | 0.11587915 | -0.680556658 |
| 1794 | -0.53875564 | -0.734523245 | -1.180331764 |
| 1795 | -0.9032578 | -0.915708635 | -1.35161619 |
| 1796 | 0.53380244 | 0.013597075 | -0.772064776 |
| 1797 | -0.76080868 | -0.80758187 | -1.318767122 |
| 1798 | -0.08208052 | -0.35754074 | -1.016086424 |
| 1799 | 0.25309388 | -0.307860875 | -0.901114686 |
| 1800 | -0.51780724 | -0.64393055 | -1.213180832 |
| 1801 | 1.20415124 | 0.39057958 | -0.51865768 |
| 1802 | 2.14682924 | 0.80555257 | -0.169049742 |
| 1803 | 1.1077886 | 0.17432605 | -0.567931282 |
| 1804 | -0.6309286 | -0.9069416 | -1.271839882 |
| 1805 | -0.02761468 | -0.302016185 | -0.97619827 |
| 1806 | 0.94439108 | 0.352589095 | -0.621897608 |
| 1807 | 1.57703276 | 0.562997935 | -0.375529598 |
| 1808 | -0.79013644 | -0.94200974 | -1.31642076 |
| 1809 | -1.39764004 | -1.33360397 | -1.541671512 |
| 1810 | 0.40811204 | -0.173433005 | -0.851841084 |
| 1811 | 0.19024868 | -0.167588315 | -0.922231944 |
| 1812 | -0.13654636 | -0.48027923 | -1.055974578 |
| 1813 | 0.49609532 | -0.13544252 | -0.809606568 |
| 1814 | 0.081317 | -0.354618395 | -0.985583718 |
| 1815 | 0.24471452 | -0.28155977 | -0.91753922 |
| 1816 | 0.69719996 | 0.001907695 | -0.746254794 |
| 1817 | 0.62178572 | -0.01854872 | -0.7767575 |
| 1818 | 0.26147324 | -0.25233632 | -0.912846496 |
| 1819 | -0.89906812 | -0.91863098 | -1.370387086 |
| 1820 | -0.91163716 | -0.956621465 | -1.38681162 |
| 1821 | -0.4214446 | -0.69068807 | -1.18737085 |
| 1822 | -0.22033996 | -0.471512195 | -1.095862732 |
| 1823 | -0.128167 | -0.418909985 | -1.063013664 |
| 1824 | 0.29499068 | -0.15297659 | -0.901114686 |
| 1825 | 1.53513596 | 0.732493945 | -0.429495924 |
| 1826 | 0.42068108 | 0.01067473 | -0.861226532 |
| 1827 | -0.45915172 | -0.60886241 | -1.194409936 |
| 1828 | 0.7307174 | 0.188937775 | -0.736869346 |
| 1829 | 1.03237436 | 0.36720082 | -0.633629418 |
| 1830 | 0.42906044 | -0.114986105 | -0.87061198 |
| 1831 | 0.05617892 | -0.290326805 | -1.006700976 |
| 1832 | -0.15330508 | -0.4978133 | -1.079438198 |
| 1833 | 0.500285 | -0.04777217 | -0.826031102 |
| 1834 | 1.2544274 | 0.451948825 | -0.549160386 |
| 1835 | -0.32089228 | -0.512425025 | -1.154521782 |
| 1836 | -0.1491154 | -0.49196861 | -1.079438198 |
| 1837 | 1.1706338 | 0.276608125 | -0.574970368 |
| 1838 | -0.24966772 | -0.565027235 | -1.126365438 |
| 1839 | -0.04018372 | -0.40429826 | -1.041896406 |
| 1840 | -0.5261866 | -0.66730931 | -1.231951728 |
| 1841 | -0.22871932 | -0.54457082 | -1.109940904 |
| 1842 | 0.41230172 | -0.17635535 | -0.865919256 |
| 1843 | -0.27899548 | -0.600095375 | -1.147482696 |
| 1844 | -0.07370116 | -0.47443454 | -1.072399112 |
| 1845 | 0.30337004 | -0.214345835 | -0.912846496 |
| 1846 | -0.24547804 | -0.45105578 | -1.114633628 |
| 1847 | -0.43401364 | -0.62055179 | -1.194409936 |
| 1848 | -0.79013644 | -0.92447567 | -1.325806208 |
| 1849 | -0.35022004 | -0.53288144 | -1.149829058 |
| 1850 | 0.30337004 | -0.11790845 | -0.887036514 |
| 1851 | -0.09883924 | -0.45105578 | -1.067706388 |
| 1852 | 0.36202556 | -0.23480225 | -0.887036514 |
| 1853 | 0.07293764 | -0.366307775 | -1.002008252 |
| 1854 | 0.14835188 | -0.34585136 | -0.962120098 |
| 1855 | 0.23214548 | -0.231879905 | -0.938656478 |
| 1856 | 0.17348996 | -0.3224726 | -0.96446646 |
| 1857 | -0.24128836 | -0.570871925 | -1.114633628 |
| 1858 | -1.9925746 | -1.564469225 | -1.792732246 |
| 1859 | -1.699297 | -1.383283835 | -1.682453232 |
| 1860 | -0.442393 | -0.641008205 | -1.192063574 |
| 1861 | -0.23709868 | -0.547493165 | -1.114633628 |
| 1862 | -0.41306524 | -0.59132834 | -1.17563904 |
| 1863 | -0.24128836 | -0.53872613 | -1.121672714 |
| 1864 | -0.30413356 | -0.59717303 | -1.126365438 |
| 1865 | -0.86974036 | -0.97123319 | -1.344577104 |
| 1866 | -1.30965676 | -1.207943135 | -1.532286064 |
| 1867 | -1.10017276 | -1.00045664 | -1.436085222 |
| 1868 | -1.06665532 | -1.0238354 | -1.419660688 |
| 1869 | -2.118265 | -1.663828955 | -1.834966762 |
| 1870 | -1.11693148 | -1.09397168 | -1.436085222 |
| 1871 | 0.0603686 | -0.45690047 | -0.980890994 |
| 1872 | -0.36278908 | -0.64977524 | -1.138097248 |
| 1873 | -0.15749476 | -0.48027923 | -1.051281854 |
| 1874 | -0.8194642 | -0.939087395 | -1.321113484 |
| 1875 | -0.78175708 | -0.90109691 | -1.30468895 |
| 1876 | -0.756619 | -0.86602877 | -1.29295714 |
| 1877 | -1.18815604 | -1.13488451 | -1.4806661 |
| 1878 | 0.09807572 | -0.407220605 | -0.969159184 |
| 1879 | 1.04913308 | 0.148024945 | -0.596087626 |
| 1880 | -0.17844316 | -0.53288144 | -1.05832094 |
| 1881 | -0.51780724 | -0.7316009 | -1.189717212 |
| 1882 | 0.43325012 | -0.231879905 | -0.826031102 |
| 1883 | -0.02761468 | -0.535803785 | -0.985583718 |
| 1884 | 1.47229076 | 0.346744405 | -0.408378666 |
| 1885 | 0.81032132 | -0.08284031 | -0.673517572 |
| 1886 | -0.4004962 | -0.769591385 | -1.15217542 |
| 1887 | -0.62673892 | -0.91863098 | -1.236644452 |
| 1888 | -0.94934428 | -1.09397168 | -1.365694362 |
| 1889 | -1.1965354 | -1.225477205 | -1.464241566 |
| 1890 | -0.55132468 | -0.927398015 | -1.215527194 |
| 1891 | 0.37040492 | -0.413065295 | -0.861226532 |
| 1892 | 0.09807572 | -0.553337855 | -0.973851908 |
| 1893 | -0.37535812 | -0.84265001 | -1.15217542 |
| 1894 | 0.13578284 | -0.60301772 | -0.94100284 |
| 1895 | 0.96952916 | -0.21142349 | -0.61251216 |
| 1896 | 0.48771596 | -0.45105578 | -0.807260206 |
| 1897 | 0.57569924 | -0.4393664 | -0.769718414 |
| 1898 | 0.71814836 | -0.348773705 | -0.713405726 |
| 1899 | 0.4793366 | -0.46274516 | -0.80022112 |
| 1900 | -1.0498966 | -1.28684645 | -1.407928878 |
| 1901 | -0.47591044 | -1.02968009 | -1.182678126 |
| 1902 | -0.128167 | -0.83096063 | -1.04658913 |
| 1903 | 0.46676756 | -0.524114405 | -0.807260206 |
| 1904 | 0.18186932 | -0.66730931 | -0.919885582 |
| 1905 | 0.17767964 | -0.71991152 | -0.910500134 |
| 1906 | 0.77680388 | -0.39845357 | -0.664132124 |
| 1907 | 1.82422388 | 0.159714325 | -0.234747878 |
| 1908 | 1.24185836 | -0.185122385 | -0.464691354 |
| 1909 | -0.7775674 | -1.172874995 | -1.274186244 |
| 1910 | -0.48010012 | -1.09981637 | -1.154521782 |
| 1911 | -0.04856308 | -0.833882975 | -0.969159184 |
| 1912 | -0.17844316 | -0.857261735 | -1.0113937 |
| 1913 | 0.0603686 | -0.73744559 | -0.901114686 |
| 1914 | 0.64273412 | -0.39260888 | -0.64770759 |
| 1915 | -0.8404126 | -1.190409065 | -1.248376262 |
| 1916 | 0.60921668 | -0.407220605 | -0.638322142 |
| 1917 | -0.7356706 | -1.11735044 | -1.180331764 |
| 1918 | -2.47857748 | -2.008665665 | -1.910050346 |
| 1919 | -1.32641548 | -1.46218715 | -1.414967964 |
| 1920 | 0.15254156 | -0.70822214 | -0.769718414 |
| 1921 | -0.95353396 | -1.225477205 | -1.229605366 |
| 1922 | -0.63511828 | -0.985844915 | -1.060667302 |
| 1923 | 0.77680388 | -0.21726818 | -0.420110476 |
| 1924 | 1.38430748 | 0.101267425 | -0.129161588 |
| 1925 | 0.27823196 | -0.50950268 | -0.593741264 |
| 1926 | 0.16092092 | -0.54457082 | -0.638322142 |
| 1927 | 1.89544844 | 0.495784 | 0.199329092 |
| 1928 | 1.81584452 | 0.40811365 | 0.192290006 |
| 1929 | 0.96533948 | -0.033160445 | -0.183127914 |
| 1930 | -0.74404996 | -0.97123319 | -0.973851908 |
| 1931 | 1.69015412 | 0.440259445 | 0.22044635 |
| 1932 | 1.5896018 | 0.422725375 | 0.20871454 |
| 1933 | 0.61340636 | -0.144209555 | -0.246479688 |
| 1934 | -0.59322148 | -0.86018408 | -0.818992016 |
| 1935 | -0.27061612 | -0.617629445 | -0.638322142 |
| 1936 | 0.50866436 | -0.12959783 | -0.220669706 |
| 1937 | 0.59245796 | -0.03608279 | -0.145586122 |
| 1938 | 0.81032132 | 0.130490875 | 0.002234684 |
| 1939 | 0.92763236 | 0.20939419 | 0.093742802 |
| 1940 | -0.47591044 | -0.59132834 | -0.631283056 |
| 1941 | 0.25309388 | -0.10621907 | -0.208937896 |
| 1942 | 1.11197828 | 0.43149241 | 0.293183572 |
| 1943 | 1.03237436 | 0.43149241 | 0.286144486 |
| 1944 | -0.07789084 | -0.2055788 | -0.305138738 |
| 1945 | 0.37040492 | 0.08081101 | -0.028268022 |
| 1946 | 1.5267566 | 0.9049123 | 0.682679664 |
| 1947 | -0.01923532 | -0.12375314 | -0.17843519 |
| 1948 | -1.07503468 | -0.80173718 | -0.781450224 |
| 1949 | -0.06951148 | -0.074073275 | -0.157317932 |
| 1950 | 0.32850812 | 0.29706454 | 0.119552784 |
| 1951 | 0.64273412 | 0.52500745 | 0.344803536 |
| 1952 | 0.34945652 | 0.329210335 | 0.187597282 |
| 1953 | 0.8354594 | 0.656512975 | 0.499663428 |
| 1954 | -0.69796348 | -0.39260888 | -0.408378666 |
| 1955 | 0.70557932 | 0.65359063 | 0.504356152 |
| 1956 | -0.41725492 | -0.144209555 | -0.194859724 |
| 1957 | 0.31593908 | 0.39642427 | 0.31430083 |
| 1958 | 0.16930028 | 0.26784109 | 0.234524522 |
| 1959 | -0.46753108 | -0.167588315 | -0.150278846 |
| 1960 | -0.36697876 | -0.00685934 | -0.051731642 |
| 1961 | 0.67206188 | 0.767562085 | 0.673294216 |
| 1962 | 1.23766868 | 1.27897246 | 1.097985738 |
| 1963 | 0.13578284 | 0.440259445 | 0.379998966 |
| 1964 | -0.89068876 | -0.325394945 | -0.323909634 |
| 1965 | -0.11140828 | 0.282452815 | 0.260334504 |
| 1966 | 0.5631302 | 0.7880185 | 0.757763248 |
| 1967 | 0.99885692 | 1.205913835 | 1.1003321 |
| 1968 | 1.52256692 | 1.708557175 | 1.515638174 |
| 1969 | 0.36621524 | 0.808474915 | 0.717875094 |
| 1970 | -0.35440972 | 0.25030702 | 0.213407264 |
| 1971 | 0.3326978 | 0.720804565 | 0.741338714 |
| 1972 | 0.55475084 | 0.95751451 | 0.93608676 |
| 1973 | 0.21957644 | 0.767562085 | 0.715528732 |
| 1974 | -0.41725492 | 0.23277295 | 0.248602694 |
| 1975 | -0.14073604 | 0.495784 | 0.50200979 |
| 1976 | -2.28166252 | -1.243011275 | -1.124019076 |
| 1977 | -1.25100124 | -0.39260888 | -0.333295082 |
| 1978 | -0.3585994 | 0.27368578 | 0.370613518 |
| 1979 | 0.03104084 | 0.67696939 | 0.692065112 |
| 1980 | 1.21672028 | 1.661799655 | 1.649380808 |
| 1981 | 0.95696012 | 1.40755564 | 1.46401821 |
| 1982 | 0.48352628 | 1.06271893 | 1.119102996 |
| 1983 | -0.68539444 | 0.118801495 | 0.204021816 |
| 1984 | 0.09807572 | 0.76463974 | 0.832846832 |
| 1985 | 0.22376612 | 0.872766505 | 0.954857656 |
| 1986 | -0.24547804 | 0.51916276 | 0.586478822 |
| 1987 | 0.3745946 | 1.06271893 | 1.105024824 |
| 1988 | 0.88573556 | 1.445546125 | 1.539101794 |
| 1989 | -1.29289804 | -0.34585136 | -0.21363062 |
| 1990 | -0.72729124 | 0.077888665 | 0.236870884 |
| 1991 | 0.3745946 | 1.024728445 | 1.144912978 |
| 1992 | -1.95905716 | -0.97123319 | -0.75329388 |
| 1993 | -0.51780724 | 0.28537516 | 0.41988712 |
| 1994 | 0.11902412 | 0.831853675 | 0.978321276 |
| 1995 | -1.63226212 | -0.62055179 | -0.469384078 |
| 1996 | -1.74538348 | -0.76082435 | -0.558545834 |
| 1997 | 0.18186932 | 0.855232435 | 1.011170344 |
| 1998 | -1.1546386 | -0.278637425 | -0.08458071 |
| 1999 | -0.63511828 | 0.188937775 | 0.356535346 |
| 2000 | -2.6838718 | -1.523556395 | -1.330498932 |
| 2001 | -0.73986028 | 0.101267425 | 0.239217246 |
| 2002 | -0.39630652 | 0.428570065 | 0.523127048 |
| 2003 | -0.5680834 | 0.241539985 | 0.394077138 |
| 2004 | -1.175587 | -0.307860875 | -0.122122502 |
| 2005 | -0.6099802 | 0.060354595 | 0.328379002 |
| 2006 | -1.05827596 | -0.255258665 | -0.04938528 |
| 2007 | 0.47514692 | 0.954592165 | 1.168376598 |
| 2008 | 0.19024868 | 0.720804565 | 0.919662226 |
| 2009 | -0.6309286 | 0.089578045 | 0.265027228 |
| 2010 | -0.66863572 | 0.05158756 | 0.182904558 |
| 2011 | -2.05123012 | -1.143651545 | -0.919885582 |
| 2012 | -0.99962044 | -0.377997155 | -0.13150795 |
| 2013 | 0.16092092 | 0.481172275 | 0.741338714 |
| 2014 | 0.86059748 | 1.094864725 | 1.191840218 |
